# A minimal model of working memory in neural systems and neuromorphic circuits

**DOI:** 10.1038/s41467-026-75704-3

**Published:** 2026-08-17

**Authors:** Damien Depannemaecker, Adrien d’Hollande, Gabriele Casagrande, Jiaming Wu, Marcelo J. Rozenberg

**Affiliations:** 1 Aix Marseille Université, INSERM, Institut de Neurosciences des Systèmes (INS), Marseille, France; 2 Université Paris-Saclay, CNRS, Laboratoire de Physique des Solides, Orsay, France; 3 Université Paris-Cité, CNRS, Integrative Neuroscience and Cognition Center, Paris, France; 4 Department of Physics, University of California, San Diego, CA USA

**Keywords:** Computational neuroscience, Electronics, photonics and device physics

## Abstract

Phenomenological spiking neuron models such as Izhikevich, adaptive quadratic integrate-and-fire (aQIF), and Adaptive Exponential (AdEx) are widely used because of their simplicity and numerical efficiency. These models reproduce diverse neuronal dynamics through a slow self-inhibitory adaptation variable. Here we introduce their symmetric counterpart by replacing adaptation with slow self-excitation, motivated by intrinsic calcium-mediated membrane currents. This minimal modification enables robust persistent spiking and working-memory dynamics without compromising computational efficiency. These properties remain in excitatory spiking neural networks. We then derive and validate a mean-field neural mass model that remains stable while retaining working-memory functionality. Additionally, we implement the single-neuron model in a minimal memristor-based neuromorphic circuit and experimentally confirm its dynamics. These results provide scalable tools for large-scale brain simulations and neuromorphic applications in robotics, brain-machine interfaces, and edge AI devices.

## Abstract (web summary)

The minimal requirements for working memory in neurons remain debated. Here, authors show that reversing the sign of a single slow current, turning adaptation into self-excitation, generates stable, tunable persistent activity, preserved at the population level and in a simple neuromorphic circuit.

## Introduction

In neuroscience, dynamical memory, also called working memory, is a basic unit of cognitive behavior. It concerns the neural circuit property of keeping a spiking state across time after its triggering stimulus terminates. This cognitive function was originally revealed in experiments where a monkey was briefly presented with a stimulus, and interrogated after a delay period during which the stimulus was no longer present. The monkey had to “keep in mind” the information in order to complete the task. In fact, during that period it was observed an increased and sustained firing rate in some prefrontal cortex neurons^1–4^.

It is intuitive that such a phenomenon of persistence can occur as a reverberating state in a neural circuit, which would require some form of feed-back loop to keep the state self-sustained. However, from a modeling perspective this is a challenge, as one needs to demonstrate two key features: that the self-excitation does not lead to runaway of the spiking rate and that the persistent state is stable against perturbations, also called ”distractors”. Consistent with this intuition, the basic models of persistent activity are though to require a neural network composed of a population of neurons with mutual excitation, which needs to be counterbalanced by a population of inhibitory neurons. The former would assure the reverberation and the later would prevent the runaway and provide stability. In fact, such models of working memory (or bi-stability) using spiking neural networks have been proposed more than 20 years ago^5–10^ and discussions can be found in most books of theoretical neuroscience^11–13^. More recent work has been described in refs. ^14,15^, among others. New mechanisms of persistent activity have been proposed, such as based on short term synaptic plasticity, and a certain debate remains, which is outside the scope of the present work^15,16^.

Focusing on the classic approaches of working memory based on recurrent excitation, there is an interesting question that also led to significant discussions. Namely, what is the minimal substrate for a working memory function. More specifically, whether a single neuron alone can implement a stable persistent state. This question has been considered both experimentally^17–19^ and theoretically^7,20–22^. For experiments, this issue may not yet be fully settled. In contrast, regarding theory, several models and mechanisms have been proposed to provide a positive answer to the question^23^. Some prominent examples are plateau potentials^12,17^, slow excitatory synapses (NMDA)^7^ and Ca-ion channel dynamics^24^.

Those relevant works have the common feature that they aimed for a mechanistic biophysical description. Indeed, they all consider biophysical spiking neuron models, which included several conductance channels and, in some cases, a multi-compartment description^5,12^. Detailed biophysical description comes at the expense of numerical efficiency, as was noted by Izhikevich^25,26^, who introduced a phenomenological model that enabled large scale numerical simulations of the cortex^27^. Shortly after, another phenomenological model was introduced by Brette and Gerstner, called Adaptive Exponential (AdEx) model^28^. They both share the feature of a slow (adaptive) variable, but the latter was argued to have a more biophysical non-linearity for the excitation, without paying a price in numerical efficiency. Despite their lack of detailed description of underlying biophysical mechanisms, one cannot emphasize enough the relevance that these phenomenological models have in Computational Neuroscience. They have become standard^11^ and the original papers are cited thousands of times. Moreover, AdEx is the spiking neuron model that is implemented in the state-of-the-art neuromorphic chip Dynapse, developed by Indiveri et al.^29,30^.

In this context, a natural question is to ask how the phenomenological models describe persistent spiking activity, which is at the heart of the working memory cognitive function discussed before. In fact, a numerically efficient model would be relevant for large-scale modeling of, for instance, epilepsy^31^, distributed working memory^32^ and the cortex^33^. However, to the best of our knowledge, there is no phenomenological single-neuron model that describes in a numerically efficient manner a working memory function. Here, we shall consider this question and demonstrate that a minimal variation on the Izhikevich, aQIF, and AdEx models, namely a *single sign-change*, provides such models. The sign change physically corresponds to transform the slow self-adaptation variable into a slow self-excitation. Our finding is rather remarkable since all three Izhikevich, aQIF, and AdEx models have been intensively used for more than 20 years now. We shall show that such a minimal working memory model can be cast as a geometrical problem, which reveals the generality of our finding. We exploit this observation to show that the adaptive Quadratic Integrate and Fire (aQIF) model can also be transformed to provide a minimal dynamical memory function. This is important as for a network of aQIF neurons it is possible to obtain an exact mean-field reduction^34^, which can be used in “whole-brain” large-scale simulations^35^. We derive such a neural mass model and demonstrate that it quantitatively reproduces numerical simulations of a population of solely excitatory spiking neurons which, contrary to common wisdom, exhibits stable persistent activity and robustness against perturbations (distractors). Finally, we extend our working memory model formulation to actual electronic hardware. We build and measure an electronic neuromorphic circuit of extreme simplicity. The circuit exploits the concept of a memristor device^36^ which implements a voltage-gated conductance^37^ similar to Hodgkin–Huxley type models. The spiking neuron is supplemented by a self-excitatory current^38^, whose simple circuit implements the form of the differential equation of the slow variable in the theoretical models. This approach, that we term “hardware modeling” opens the way to seamlessly port theoretical spiking neural networks of a low dimensionality into their hardware counterparts. This may find useful applications in brain-machine-interfaces, robotics and edge AI medical devices.

## Results

### From adaptation to self-excitation

We begin with the general form of the phenomenological models of spiking neurons with adaptation, which are defined by two differential equations plus a reset condition^11,39^.

The equations read,

1 $$C\frac{dV}{dt}=-{g}_{L}V+F(V)-{I}_{w}+{I}_{ext}$$

2 $${\tau }_{w}\frac{d{I}_{w}}{dt}=-{I}_{w}+H(V)$$

where *V* denotes the membrane potential of the neurons, *C* is the capacitance and *g*_*L*_ the leak conductance. *I*_*ext*_ is the input excitatory current and *I*_*w*_ denotes the intrinsic membrane current. *F*(*V*) is a non-linear function of *V* which captures the spike initiation phenomenon and *H*(*V*) specifies the coupling of the *I*_*w*_ slow variable equation with that of the membrane potential. In these models, the initiation of the spike occurs when the function *F*(*V*) becomes positive enough to overcome the leak term, causing a runaway of the membrane potential *V*(*t*). Hence, an ad hoc cut-off needs to be introduced, which is known as the reset condition and reads,

3 $${{{\rm{if}}}}\quad V={V}_{spike}\quad {{{\rm{then}}}}\quad V\leftarrow {V}_{reset}$$

where *V*_*spike*_ and *V*_*reset*_ are parameters of the model. In addition, the emission of each spike has an effect on the intrinsic current *I*_*w*_ described by (2). When *V* reaches *V*_*spike*_ the current *I*_*w*_ is increased in an amount given by the model parameter *b*,

4 $${{{\rm{if}}}}\quad V={V}_{spike}\quad {{{\rm{then}}}}\quad {I}_{w}\leftarrow {I}_{w}+b$$

This last expression completes the model, which is thus defined by the system (1)–(4).

The two most popular models of this form are Izhikevich’s^25^ and AdEx^28^. This minimal formulation sacrifices the detailed biophysical description for numerically efficiency. Nevertheless, they keep the diversity of biologically relevant dynamical behaviors^25–27^. We should also include here the aQIF model, which consists in the Quadratic Integrate and Fire model^40^ augmented with a slow adaptation variable^41^. The relevance of the aQIF stems from the fact that an exact mean-field reduction of a fully connected network of these neurons can be obtained analytically, providing a useful tool for the study of large-scale whole-brain neural networks^34,42^. We note that in all three models, aQIF, Izhikevich and AdEx, the second equation for the slow variable (2) has the same simple form, where the function *H*(*V*) is linear. Nevertheless, it can also be non-linear, such as in the Conductance-Based Adaptive Exponential Integrate-and-Fire model (CAdEx)^43^.

The difference between these three models is the definition of the non-linear function *F*(*V*), in the equation of the membrane potential (1). In the aQIF model *F*(*V*) is a simple quadratic polynomial, while in Izhikevich the coefficients of the polynomial are chosen to numerically match measurements in biological neurons. In the AdEx, *F*(*V*) is an exponential function, which provides a better fit to measured spike traces of neurons^11^.

In all these models, the slow variable describes an inhibitory intrinsic membrane current that produces adaptation. This is encoded in the negative sign of the current *I*_*w*_ in Eq. (1).

However, as discussed in the introduction, a dynamical memory requires excitatory recurrence to achieve persistent spiking. This motivates us to extend the above neuron model by turning the adaptation variable into a self-excitatory one, which is simply done by flipping the *single sign* of the *I*_*w*_ term of Eq. (1). It now reads,

5 $$C\frac{dV}{dt}=-{g}_{L}V+F(V)+{I}_{w}+{I}_{ext}$$

6 $${\tau }_{w}\frac{d{I}_{w}}{dt}=-{I}_{w}+H(V)$$

This change is also biologically motivated. Excitatory membrane current exist in biological neurons, such as the transient inward Ca2+ ionic current, known as *I*_*T*_. The relevance of Ca currents in the context of biorealistic single neuron models of persistent behavior has been discussed by Fransen et al.^24^.

Moreover, the fact that the change involves *I*_*w*_, which is the slow variable, is consistent with the biophysical model of working memory introduced by Wang^7^. In fact, a key contribution of that work was to emphasize that a stable persistent spiking state requires the recurrent excitation to be a slow current component (NMDA).

In the following, we shall explore the behavior of the presently introduced (self-)excitatory version of these three popular theoretical models. They are defined by Eqs. (5) and (6), supplemented by Eqs. (3) and (4). For definiteness, we adopt below the *F*(*V*) of the aQIF model, which we shall also use again later for the derivation of a neural mass model.

We describe the extensions of Izhikevich and AdEx models in Supplementary Information, and show that they exhibit the same qualitative properties.

**Fig. 1:**
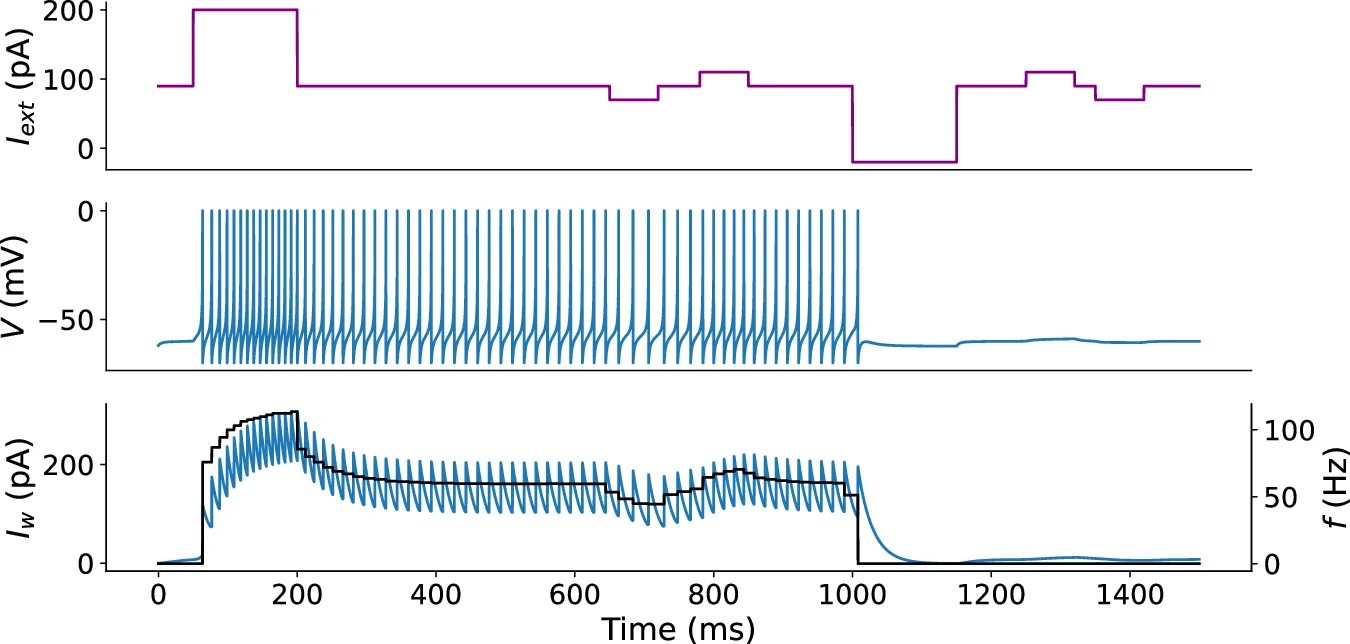
Dynamical memory attractor in the eQIF model. The traces show the behavior of the dynamical memory and its stability against perturbations. Initially, the neuron is quiet as *I*_*ext*_ is subthreshold. A first pulse at 100 ms drives the neuron to the dynamical attractor. After *t* = 200 ms the spiking persists although the *I*_*ext*_ is subthreshold. At *t* = 700 ms a sequence of perturbation pulses (distractor) is applied, but the systems returns to the persistent state. At *t* = 1000 ms an inhibitory pulse is applied and the spiking stops. The neuron remains quiescent when *I*_*ext*_ returns to the subthreshold value at *t* = 1100 ms, demonstrating bistability. The quiescent state is also stable under a new distractor at *t* = 1300 ms. In the bottom panel we highlight the approximate proportionality between instantaneous frequency (black) and feedback current *I*_*w*_ (blue).

The model equations of the extension of the adaptive-QIF to the self-excitatory model case (that we term eQIF) read,

7 $$C\frac{dV}{dt}={g}_{L}({E}_{L}-V)({V}_{T}-V)+{I}_{w}+{I}_{ext}$$

8 $${\tau }_{w}\frac{d{I}_{w}}{dt}=a(V-{E}_{L})-{I}_{w}$$

9 $${{{\rm{if}}}}\quad V={V}_{spike}\quad {{{\rm{then}}}}\\ \; V \!\! \leftarrow {V}_{reset}\quad {{{\rm{and}}}}\quad {I}_{w}\leftarrow {I}_{w}+b$$

where *a*, *E*_*L*_ and *V*_*T*_, *V*_*spike*_, *V*_*reset*_ and *b* are parameters of the model. The precise parameter values for the different simulations of the eQIF model are presented in III C. The only difference with respect to the aQIF is the change of sign of the term *I*_*w*_ in (7). In Fig. 1, we illustrate the basic cognitive function of the model, namely, the stable working memory (for similar results in the Izhikevich and AdEx see Supplementary Information). The stability is demonstrated by the fact that both states, persistent and quiescent, are robust against *distractor* perturbations in the input (at *t* = 700ms and 1300 ms).

**Fig. 2:**
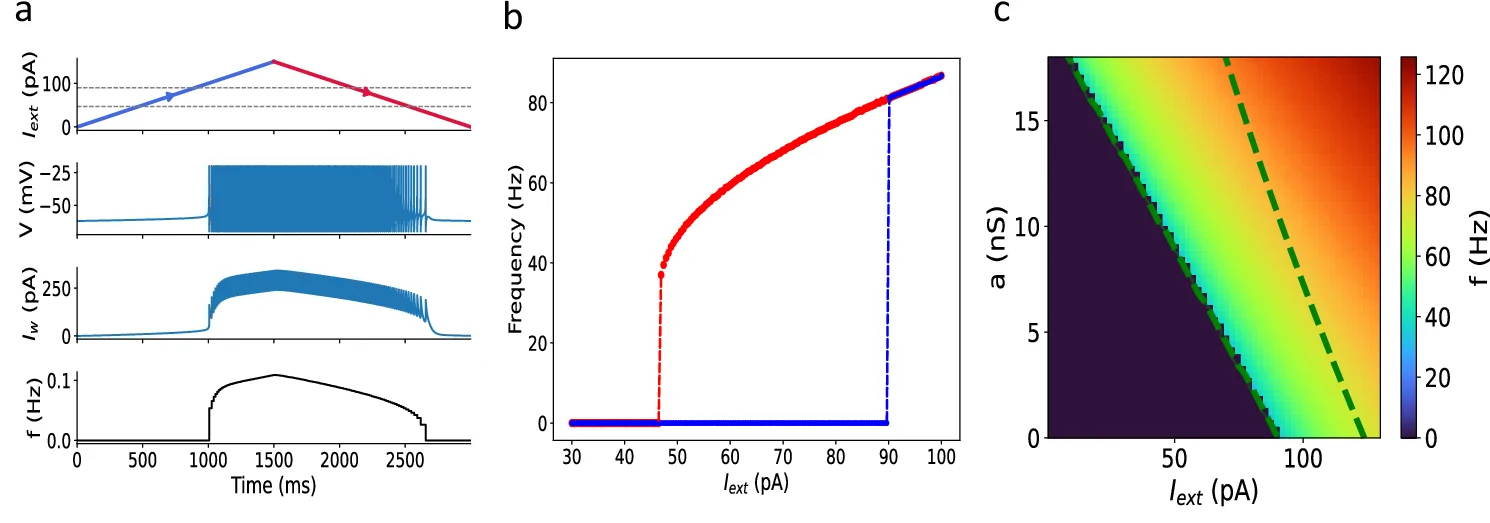
Hysteresis behavior of the eQIF model. **a** Response of the eQIF model when it is excited by a gradually increasing (blue) and then decreasing (red) input current *I*_*ext*_(*t*) shown on top. Thin black lines indicate the spiking onset and offset currents. The hysteresis region is in between them. Below, the tonic spiking action potentials *V*(*t*) and the feedback membrane current *I*_*w*_(*t*), which shows a rapid variation. Its mean value follows the spiking frequency *f*(*t*) that is shown in the bottom panel. **b** Large hysteresis in the response function *f*(*I*_*ext*_) obtained increasing (blue) and decreasing (red) external input current *I*_*ext*_(*t*). **c** Amplitude of the bistability region (within the green doted lines) is reported for different values of parameter *a* around the value 10 nS used in (**a**). We fixed the after-spike jump *b* = 100 pA.

To clearly illustrate this point, we turn to the response function of the model. The bistability region, where the working memory functionality is realized, shows up in the response function as hysteresis. This can be seen in the results of Fig. 2a where we gradually increase the applied current *I*_*ext*_ and then decrease it. A large hysteresis loop forms in the response (Fig. 2b).

Interestingly, the behavior of our minimal model bears qualitative similarity with the experimental recordings of a squid axon by Guttman et al.^44^ and to the simulations of Booth et al.^45^, obtained in a complex biophysical multi-compartment model.

Importantly, the parameter region where bistability can be realized is large and does not require fine tuning. We evaluate this region of bistability for a large range of value of parameter *a* for different *I*_*ext*_ in Fig. 2c.

Changing the value of parameter *b* (i.e. the after-spike jump in *I*_*w*_) affects the output spiking frequency, which increases with *b* at fixed external current *I*_*ext*_ (see Supplementary Fig 1a in Supplementary Information).

### Nullcline and bifurcation

The dynamics described previously can be formally understood by considering the topology of the phase plane.

**Fig. 3:**
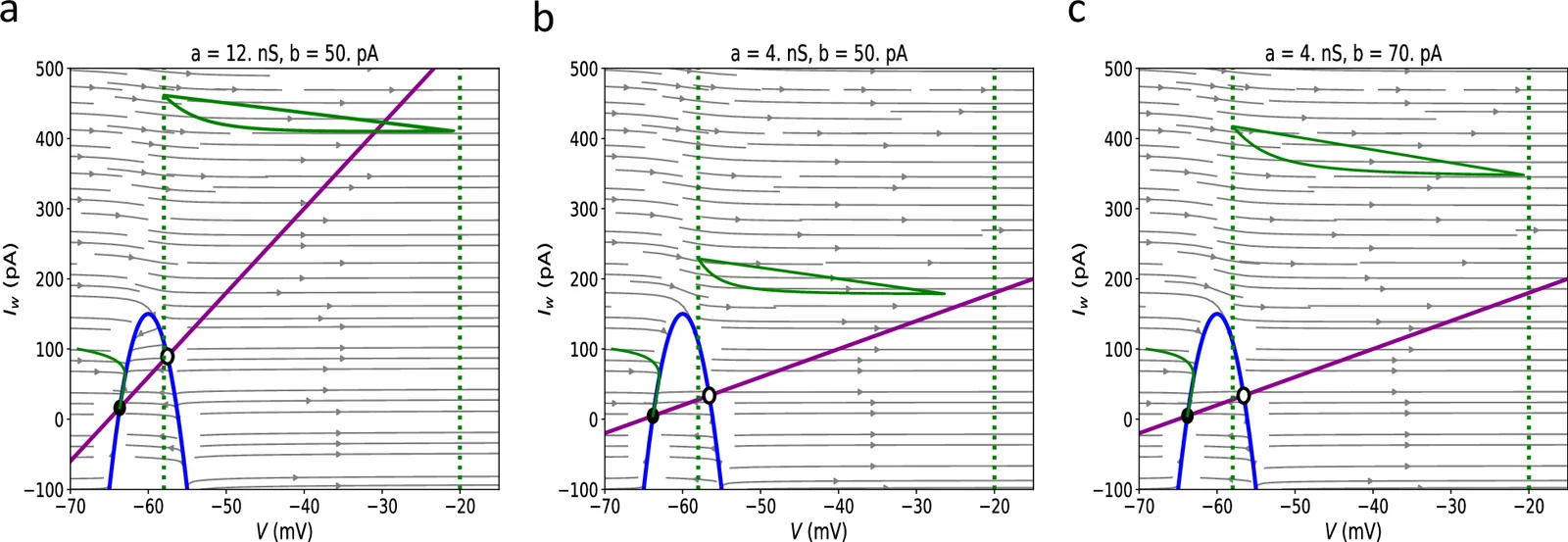
Phase-plane of the eQIF model for *I*_*ext*_ = 100 pA. The *V*-nullcline (inverted parabola) is shown in blue and the *I*_*w*_-nullcline in purple. Their intersection defines two fixed-points, a stable one (black) associated with resting state and an unstable one (white). On the right side of the right branch of the *V*-nullcline the flow diverges toward the spiking threshold (−20 mV) shown by a green dashed line. The reset potential (−58 mV) is also shown by a green dashed line. Representative trajectories for two different initial conditions are plotted in green: one converges to the resting state, while the other generates a stable spiking cycle. From panel (**a**) to panel (**b**), the parameter *a* is varied. From panel (**b**) to panel (**c**), the parameter *b* is modified. Since *b* remains positive, the reset mechanism re-injects trajectories into a region of phase space that can sustain repeated spiking. In particular, when the reset occurs to the left of the right branch of the *V*-nullcline, trajectories may still converge to the resting fixed point; otherwise, sustained spiking persists. This mechanism explains the robustness of stable spiking behavior over a broad region of parameter space.

In Fig. 3 we show the linear *I*_*w*_-nullcline and the *V*-nullcline, which has the quadratic form of the non-linearity of this model, for an external input *I*_*ext*_ = 100 pA. The parabola is flipped upside down with respect to the usual case (AdEx, Izhikevich), since the sign of *I*_*w*_ is flipped in the present model (Eq.(7)).

The crossings of the nullclines indicate the existence of two fixed points: one stable corresponding to the resting state, and one unstable on the separatrix following the right branch of the *V*-nullcline. The flow diverges from the unstable point, directing the system towards the reset threshold, indicated by a vertical green dashed line at *V* = *V*_*spike*_ = −20 mV.

Moreover, in Fig. 3, we observe the system exhibiting a bistable regime. There, both a stable (resting) fixed point and a stable periodic trajectory coexist and is the region where a working memory function is realized. From the topology of this phase plane analysis, one concludes that the region is generically large without the need of fine tuning of the model parameters.

**Fig. 4:**
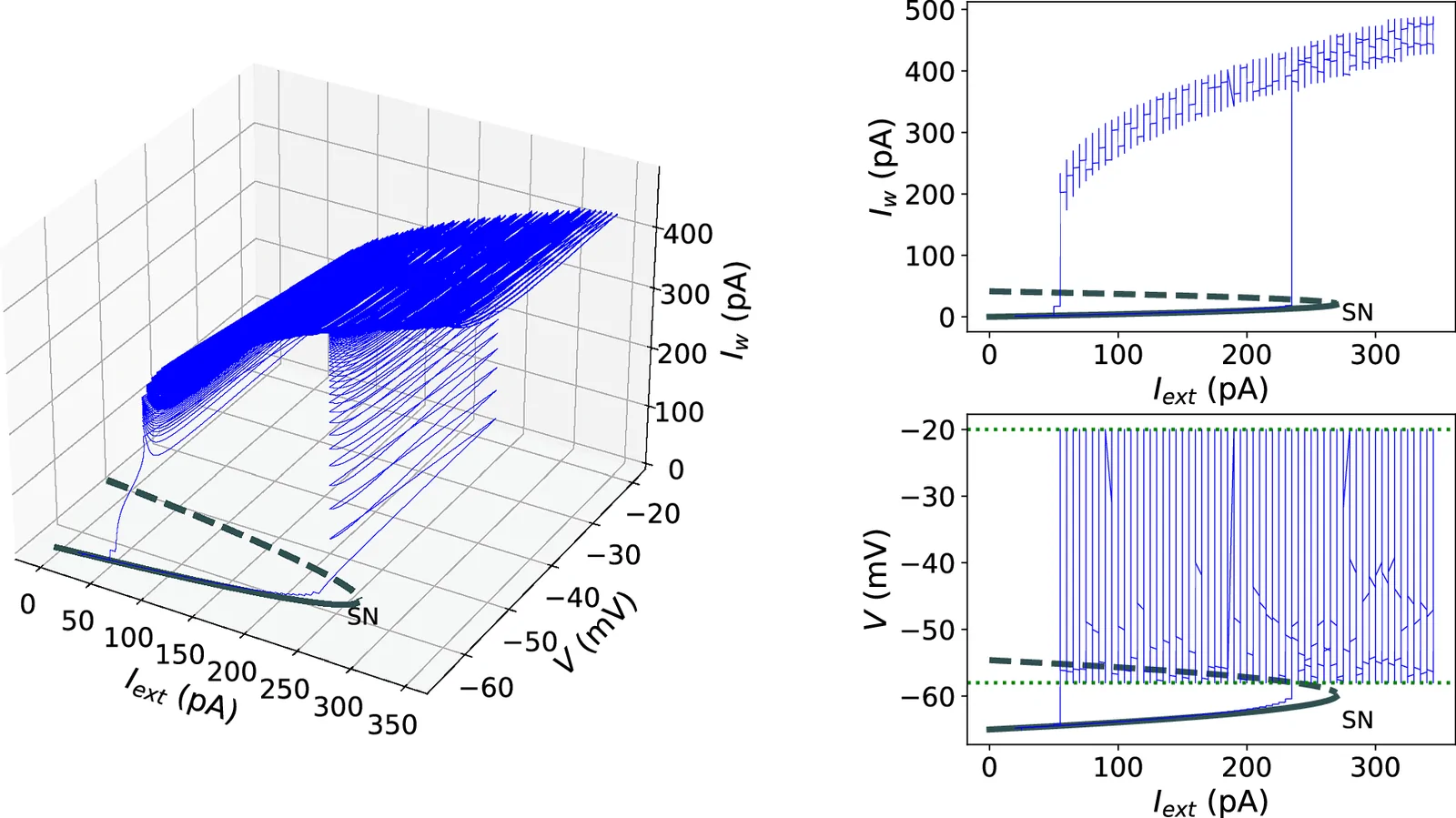
Behavior of the model variables *V* and *I*_*w*_ as the external current *I*_*ext*_ is varied up and down as in Fig. 2. On the left: Stable fixed-point (full line) and unstable fixed-point (dashed line) disappearing in a saddle-node bifurcation (SN) while increasing *I*_*ext*_. Within a range of *I*_*ext*_ the stable fixed point co-exist with a limit-cycle defined by the reset mechanisms, as shown by the simulation (in blue) for continuously increasing and then decreasing *I*_*ext*_. On the right: (top) Projection on the *I*_*ext*_-*I*_*w*_ plane, here the width of the limit-cycle (blue traces) correspond to the parameter *b*. (bottom) Projection on the *I*_*ext*_-*V* plane, where the width of the limit-cycle is constraint by *V*_*spike*_ and *V*_*reset*_ (respectively at −20 mV and −58 mV shown with green dashed lines). We note that in this case a = 4nS and b = 60pA.

To induce tonic spiking *I*_*ext*_ has to be increased, which pushes the parabola downwards. The stable fixed point slides along the parabola until it evolves into a limit cycle. The emergence of a limit cycle and the coordinates of the fixed-points can be systematically studied depending on *I*_*ext*_, as shown in Fig. 4.

At large *I*_*ext*_ a limit cycle is the only solution. The saddle-node bifurcation (denoted SN in Fig. 4) marks the critical value of *I*_*ext*_ (≳250pA) beyond which the fixed points disappear, leaving the limit-cycle as the only attractor in the system. In this purely oscillatory regime, the neuron’s dynamics are entirely dictated by this reset-induced periodic orbit, reflecting continuous tonic spiking behavior.

This bistability arises due to the nonlinear dynamics introduced by the reset condition, which effectively extends the two-dimensional phase space into a hybrid dynamical system. Indeed, this reset mechanism is equivalent to a third dimension enabling the existence of a limit-cycle, corresponding to the spiking activity. The limit-cycle does not emerge from the continuous flow of the differential equations alone, but is instead a consequence of the imposed discontinuity in the voltage dynamics. Along the *V* direction, its location is fixed and constrained by the model parameters *V*_*spike*_ and *V*_*reset*_ (shown with green dotted lines in Figs. 3 and 4).

Along the *I*_*w*_ dimension, the width of the limit cycle depends on the model parameter *b*, and its steady state depends on the dynamics of *I*_*w*_, particularly on the time constant *τ*_*w*_.

The presence of this artificial, yet physiologically motivated, reset condition ensures that the system cannot settle at the unstable fixed point once the threshold is crossed. Instead, it follows a repeatable path that mimics sustained spiking activity. This hybrid structure, a continuous flow interrupted by instantaneous transitions, enables oscillatory dynamics even in the absence of intrinsic limit-cycle behavior in the underlying continuous system.

### Analog hardware implementation using a memristive spiking neuron

**Fig. 5:**
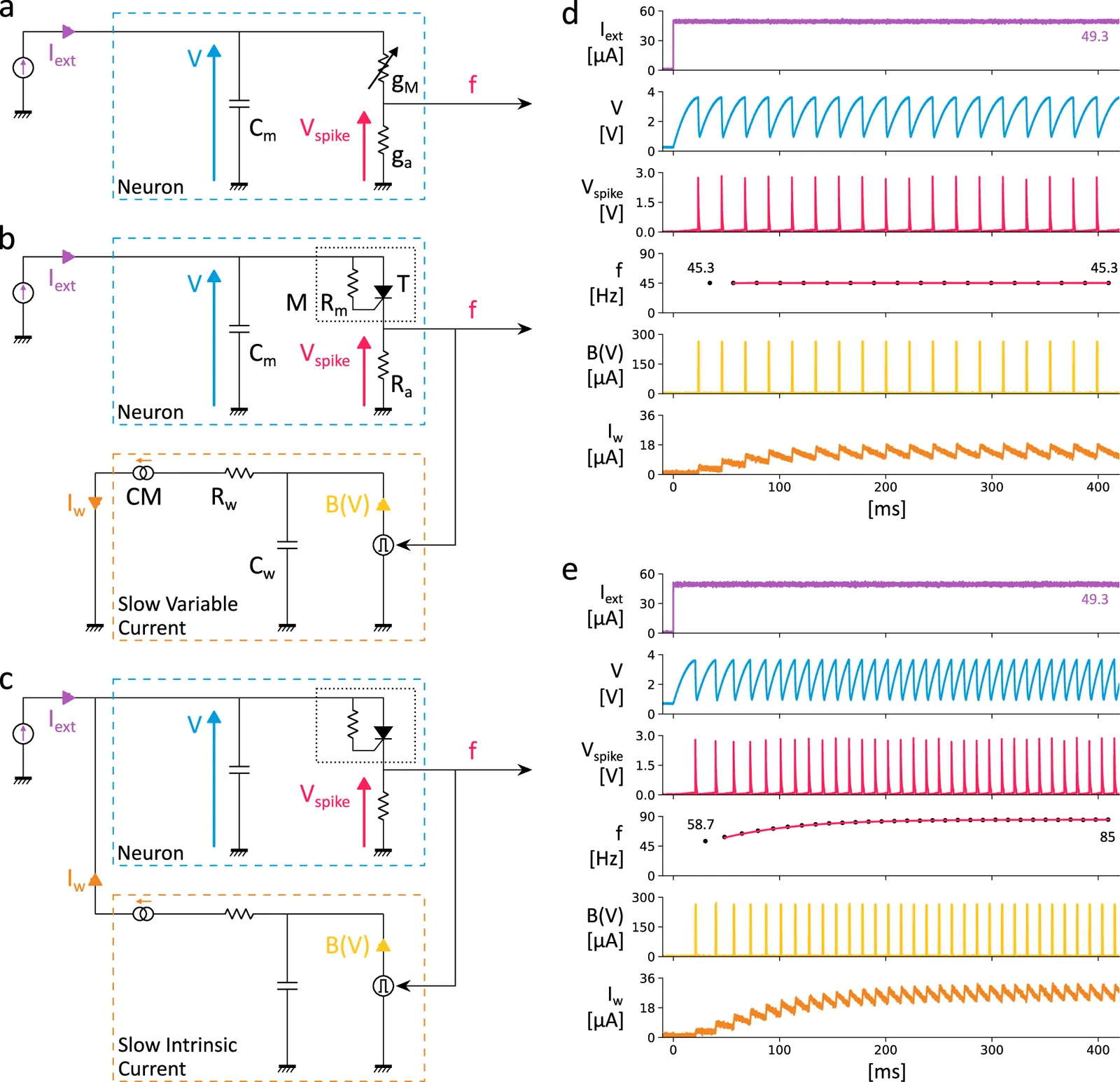
Electronic implementation of a minimal spiking neuron model of working memory. On the left, the hardware schematics: **a** Memristor-based analog-LIF neuron (aLIF), **b** open-loop configuration (no working memory function), and **c** close-loop configuration (the hardware model with a working memory function). On the right, the corresponding traces in response to a step function input current are shown in (**d**) and (**e**), for the open-loop and closed-loop configurations, respectively. From top to bottom, the external current *I*_*ext*_, the membrane voltage *V*, the spiking voltage *V*_*spike*_, the firing rate *f*, estimated from interspike intervals (black dots) and by Gaussian-kernel-smoothing (red line), the current pulses *B*(*V*), and the slow variable intrinsic current *I*_*w*_. Both circuit schematics contain two blocks: the aLIF neuron (top blue box) and the slow variable current *I*_*w*_ (bottom orange box). The aLIF neuron takes an input current *I*_*ext*_ (≈49.3 μA) charging the capacitance *C*_*m*_ to a membrane voltage *V*. When *V* exceeds a threshold, *C*_*m*_ discharges through the resistor *R*_*a*_, resetting *V*, and the neuron produces a spiking response (*V*_*spike*_). Each spike triggers a current pulse *B*(*V*) that feeds into the slow variable *I*_*w*_. In the open-loop configuration (**b**, **d**), *I*_*w*_ is routed to ground and therefore does not affect the spiking. In the closed-loop configuration (**c**, **e**), *I*_*w*_ is fed back into the neuron, adding to *I*_*ext*_, and increasing the spiking frequency response within a delay  ~ *τ*_*w*_ (22 ms, *τ*_*w*_ = *R*_*w*_*C*_*w*_). The conductance *g*_*M*_ in Eq.(10) corresponds to the two-terminal memristor in the dotted-line box M. The hardware parameters are *C*_*m*_ = 220 nF, *R*_*m*_ = 680 k*Ω*, *R*_*a*_ = 2.2 k*Ω*, *C*_*w*_ = 2.2 μF, *R*_*w*_ = 10 k*Ω*, using a P0118MA thyristor (T) (see Supplementary Information for circuit details).

In order to demonstrate the generality of our theoretical model, we shall now show how the idea can also be exploited to implement actual neuromorphic hardware. To this end, we adopt the analog neuromorphic circuits of unprecedented simplicity that were introduced recently^38^. The circuits are schematically shown in Fig. 5, where the neuron and synapse blocks are shown (for detailed circuit components and values see Supplementary Information). The two blocks can be considered actual physical embodiments of the two respective Eqs. (5) and (6).

**Fig. 6:**
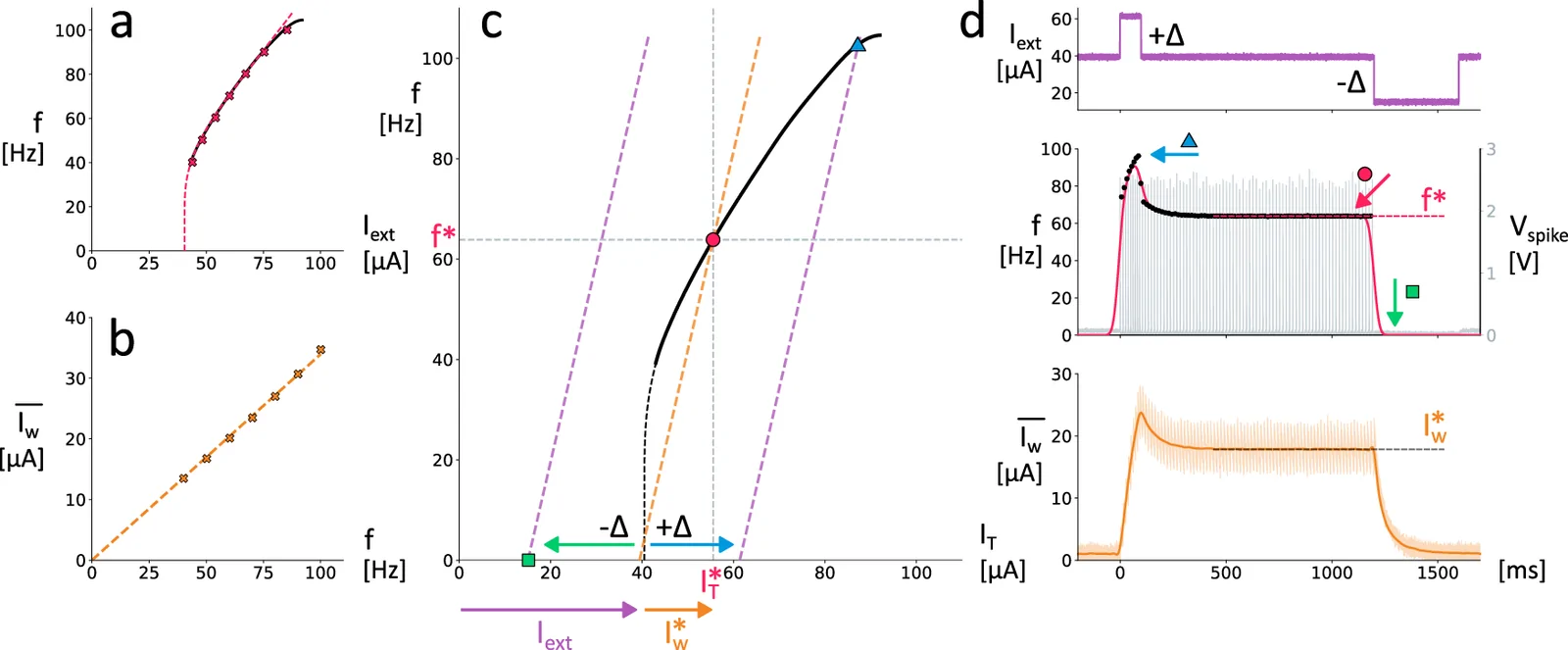
Geometric construction of the self-consistent solution. **a** Measured spiking frequency response as function of input current *f*(*I*_*ext*_) in open-loop, i.e. *I*_*ext*_ = *I*_*T*_, indicated by red-crosses. In red dashed-line is the fit with the LIF response form $A/[1-\log (B(1-I)/{I}_{min})]$ with *A* and *B* constants and *I*_*min*_ the threshold value for excitation of the input current *I*_*ext*_. **b** Measured slow current as function of the spiking frequency $\overline{{I}_{w}}(f)$ in open-loop, i.e. no feed-back, indicated by orange crosses. Data show the linear relation $\overline{{I}_{w}}\approx (1/\alpha )f$ indicated by orange dashed-line. **c** Geometric construction of the self-consistent solution $({I}_{T}^{*},{f}^{*})$ indicated by the red circle. The black line is the response from (**a**), the orange dashed-line from (**b**) (flipped x-y axis and shifted by *I*_*ext*_). They correspond to the Eqs. (17) and (18), respectively. The purple dashed-lines correspond to the shifts of the orange line during the excitatory (*t* = 100 ms) and the inhibitory (*t* = 1300 ms) pulses ± *Δ*. The input pulses provoke the shift of the frequency to the blue triangle and green square (excitatory and inhibitory, respectively). **d** Time-dependent traces during the working memory function. (top) Applied protocol of input current *I*_*ext*_(*t*) with a subthreshold base-line *I*_*ext*_ = 38 μA and ±*Δ* excitatory and inhibitory pulses. (center) Measured instantaneous frequency *f*(*t*) (black dots) and spikes (gray line). The measured self-consistent value f^*^ ≈ 63Hz during the working memory is in agreement with the geometrical solution. The blue and green arrow indicate the shifts of *f* during the ±*Δ* pulses. (bottom) In light-orange the measured slow feed-back current *I*_*w*_(*t*) and its mean value $\overline{{I}_{w}}(t)$. The width of *I*_*w*_ indicates the amplitude of the current pulses *b* of *B*(*V*) as in Eqs. (13), (14) and (15). The measured self-consistent value ${\overline{{I}_{w}}}^{*}\approx 18\mu$A, gives a ${I}_{T}^{*}={I}_{ext}+{I}_{w}^{*}\approx 38\mu$A + 18 μA = 56 μA in agreement with the geometrical solution. Additional cases are shown in the Supplemental Material.

The neuron implements an analogue leaky-integrate-and-fire (aLIF) model^38^. In the circuit block one recognizes the external input current and the membrane capacitor C_*m*_ (Fig. 6). Crucially, the voltage dependent switch of the LIF model is implemented here by a *memristor* device, which consists of a thyristor (T) with a resistor (R_*m*_) connected between the anode and gate of T. The memristor (M) is a two-terminal device whose resistance (or conductance) depends on the value of the potential between its terminals. The memristor is open (low conductance) when the potential is low and close (high conductance) when the potential exceeds a certain threshold *V*_*th*_^37^. Hence, the memristor behaves as a voltage-gated conductance, which is similar to the ionic conductance channels of the Hodgkin–Huxley model, as we can see from the circuit equations below,

10 $${C}_{m}\frac{dV}{dt}=-{g}_{M}(V-{V}_{a})+{I}_{ext}$$

11 $${V}_{a}=V\frac{{R}_{a}}{{R}_{a}+1/{g}_{M}}$$

where *g*_*M*_ is a voltage-dependent conductance, which behaves as a switch in a LIF model.

12 $${g}_{M}=\left\{\begin{array}{ll}\frac{1}{{R}_{m}}\quad &\,{{\mbox{if}}}\,\,\,(V-{V}_{a}) < {V}_{th}\quad (\,{\mbox{i.e. thyristor open}})\hfill\\ \infty \quad &\,{\mbox{if}}\,\,\,(V-{V}_{a})\ge {V}_{th}\quad (\,{\mbox{i.e. thyristor closed}})\end{array}\right.$$

When the thyristor is open, the low conductance of the memristor device is set by the value of the large leak resistance *R*_*m*_. The output spike signal *V*_*spike*_ is the voltage drop *V*_*a*_ at the small resistance *R*_*a*_. Since *R*_*m*_ ≫ *R*_*a*_, from Eq. (11), one has that *V*_*a*_ = *V*_*spike*_ ≈ 0, when the thyristor is open, and a spike of height *V*_*spike*_ ≈ *V*_*th*_ emitted when the thyristor, and hence the memristor, switches to closed.

Our minimal memristive neuron model circuit shares features of two paradigmatic models: the dynamical mechanism of excitability due to a voltage-gated conduction channel, like the Hodgkin–Huxley model, and the conceptual simplicity of a LIF model, with a leaky-integration plus a non-linearity due to a switch with a threshold. An important point to emphasize is that in the LIF model the spike emission is an abstract event with a reset voltage condition *V* ← *V*_*reset*_. In contrast, in our circuit model the spike is a bona fide electric signal, hence we call our model analog-LIF (aLIF). In fact, the spike is the fast but continuous discharge of *C*_*m*_ on *R*_*a*_, hence its duration is simply given by *τ*_*spike*_ = *C*_*m*_*R*_*a*_. Adopting *C*_*m*_ = 1 μF and *R*_*a*_ = 2.2*KΩ*, *τ*_*spike*_ = 2.2 ms, similar to biological spikes.

To complete the specification of the aLIF model we can estimate the parameters *V*_*th*_ and *V*_*reset*_, which are respectively obtained from the information in the data sheet of the thyristor device *I*_*GT*_ and *I*_*H*_. The former is the *critical gate* current, which gives *V*_*th*_ ≈ *I*_*GT*_*R*_*m*_ and the latter is the *holding* current, which gives *V*_*reset*_ ≈ *I*_*H*_*R*_*a*_.

The synapse circuit block implements the slow current variable. To see this we start with the equation that describes the circuit of the feedback current (cf. Fig. 5). It reads,

13 $${C}_{w}\frac{d{V}_{w}}{dt}=-\frac{{V}_{w}}{{R}_{w}}+B(V)$$

and replacing *τ*_*w*_ = *R*_*w*_*C*_*w*_ and *I*_*w*_ = *V*_*w*_/*R*_*w*_, we get

14 $${\tau }_{w}\frac{d{I}_{w}}{dt}=-{I}_{w}+B(V)$$

which is the model Eq. (6).

Note that term *B*(*V*) is implemented as a current source commanded by the neuron output. It injects a current pulse of intensity *b* every time there is a spike. Hence,

15 $$B[V(t)]=b{\Sigma }_{i}\delta \left(t-{t}_{i}^{f}\right)$$

where ${t}_{i}^{f}$ denote the *i*th spike event. This implements the conditions (4) and (9).

As a technical note, the current *I*_*w*_ that flows through *R*_*w*_ and is injected back to the neuron as in (5), is implemented by a current mirror (CM) indicated with a double-circle symbol. The CM has the function of copying its input to its output. This is needed to faithfully represent Eq. (14).

We can experimentally explore the basic behavior of this system by application of a constant input current *I*_*ext*_. To characterize the circuit response it is useful to consider, in addition to the feed-back case, also the open-loop circuit. This mathematically corresponds to suppressing the feed-back term +*I*_*w*_ from (5), and in the circuit it corresponds to disconnect the feedback by sending *I*_*w*_ to ground, as shown in Fig. 5.

The comparison between open- and close-loop are shown in panels d and e of Fig. 5. In the open-loop case there is no feed-back of the slow current to the neuron. Hence the neuron produces tonic spikes at a constant rate^37^. The slow *I*_*w*_ results from the integration the *b* delta-like current pulses produced by each spike (cf. Eq. (15)). The current *I*_*w*_(*t*) achieves an approximate constant value within a time ~*τ*_*w*_. In the right-most panels, we show the behavior for the close-loop, when the *I*_*w*_ is fed-back to the neuron. The total input current to the neuron is now time-dependent *I*_*T*_ = *I*_*ext*_ + *I*_*w*_(*t*). We observe that due to the feed-back, as *I*_*w*_(*t*) increases the spiking rate *f* also increases and achieves an asymptotic (self-consistent) value within a time ~*τ*_*w*_. Thus, the behavior is qualitatively opposite to the familiar adaptation, where the inhibitory feed-back reduces the spiking frequency. We should also note that similarly to the mathematical model discussed before (cf Fig. 4), *I*_*w*_(*t*) has a fast component due to the integration of the spikes, which is on top a slow variation $\overline{{I}_{w}}(t)$. Since the fast component has a relatively small amplitude, ${I}_{w}\approx \overline{{I}_{w}}$. We shall adopt this slow component in our analysis of the self-consistent state below.

It is intuitive that the feed-back may be cast as a self-consistent problem. Geometrical solutions are commonly used in the discussion of neural networks, where the states are represented by population rates that need to be determined self-consistently^12,13^. We can adopt this method for our hardware model of a single neuron with current feed-back.

To construct the solution we need the open-loop response of the system, as shown in Fig. 6. In panels a and b we have the measured functions *f*(*I*_*ext*_) and $\overline{{I}_{w}}(f)$. We note that the slow current is linear in its input *f*, hence, we have the relation

16 $$f=\alpha \overline{{I}_{w}}$$

where *α* denotes the inverse of the slope of $\overline{{I}_{w}}(f)$.

The key point is that we can write the close-loop equations in terms of the open-loop responses. The spiking rate *f* of the neuron only depends on whatever its total input current is, where *I*_*T*_ = *I*_*ext*_ + *I*_*w*_. Using this and replacing in (16), we have to solve the system below to determine the self-consistent values $({I}_{T}^{*},{f}^{*})$,

17 $${f}^{*}=f({I}_{T}^{*})$$

18 $${f}^{*}=\alpha {\overline{{I}_{w}}}^{*}=\alpha ({I}_{T}^{*}-{I}_{ext})$$

In the center panel c of Fig. 6 this system is solved geometrically. On the panels d we show the explicit time-dependent behavior of all quantities during the working memory function protocol (top panel). In the center and bottom panels we can observe the dynamical evolution of the system as it moves and reaches the persistent self-consistent state. We can also observe the evolution of the behavior as the dynamical attractor is finally toggled to the resting state by the (−Δ) pulse at *t* = 1300 ms.

**Fig. 7:**
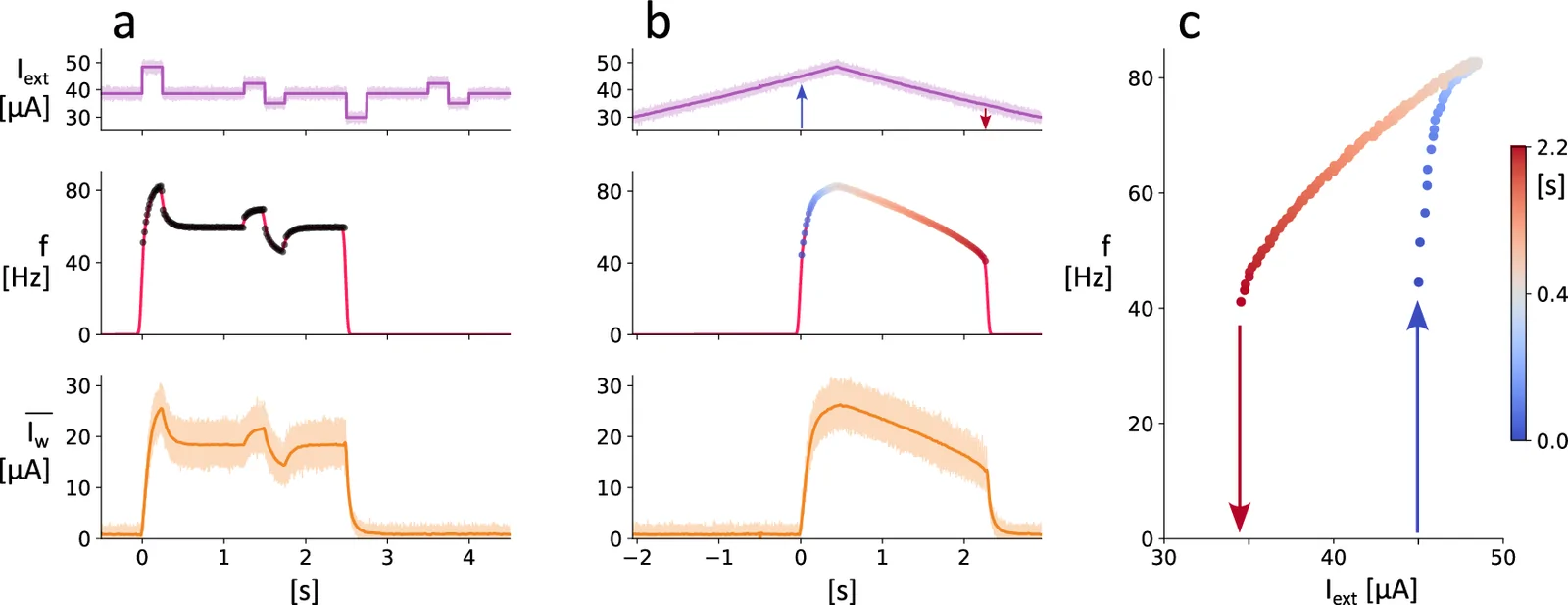
Stability and hysteresis of a hardware-implemented dynamical memory attractor. **a** Traces illustrating the resilience of the hardware dynamical memory. The electronic neuron is biased with a baseline current *I*_*ext*_ (≈38 μA, top plot). A sufficiently strong positive pulse (here to ≈48 μA) triggers spiking (rate displayed in the middle plot, estimated as in Fig. 5), which in turn drives the slow intrinsic current $\overline{{I}_{w}}$ (bottom plot) to rise and further amplifies the spiking activity. When the positive pulse ends, the neuron continues to fire, and although transient perturbations occur, the dynamics relax back to the stable spiking regime. Conversely, a sufficiently strong negative pulse (here to ≈30 μA) suppresses spiking, thereby interrupting the sustainment of the intrinsic current. After the negative pulse ends, the neuron remains quiescent and does not resume firing, even though transient perturbations may still be observed. **b** Traces illustrating the hysteresis of the same hardware implementation. The electronic neuron is driven by a triangular ramp of the input current *I*_*ext*_. Spiking onset occurs at ≈45 μA (blue upward arrow), whereas spiking cessation occurs only once *I*_*ext*_ decreases to ≈35 μA (red downward arrow). The dots (middle plot) indicate the instantaneous firing rate and are color-coded according to the direction of *I*_*ext*_: blue during the rising phase, fading to white near the maximum, and turning red during the descending phase. **c** Instantaneous firing rate as a function of the external input current *I*_*ext*_, using the same color-coded dots as in (**b**). The arrows mark the spiking onset (blue upward) and cessation (red downward) current thresholds, thereby delineating the hardware dynamical memory bistability region. The hardware is the same as in Fig. 5.

We shall now experimentally demonstrate that our hardware model provides a faithful physical embodiment of a working memory function and bistable behavior, in full analogy with the mathematical models described in previous sections. This is shown in Fig. 7.

In Fig. 7a, we excite the circuit with an input current *I*_*ext*_(*t*) that follows a similar protocol as used previously in Fig. 1. We observe that initially *I*_*ext*_ = 38 μA is subthreshold, so it is not eliciting spikes. Then, at *t* = 0 s an excitatory current pulse of duration 0.25 s, toggles the neuron from the quiescent state to the self-sustained spiking state with a stable frequency. At *t* = 1.25 s we perturb the state, to demonstrate the robust stability of the dynamical attractor, as it quickly returns to the self-consistent persistent state. Then, at *t* = 2.5 s we apply a strong inhibitory pulse, which lowers *I*_*ext*_ and stops the spiking. Thus, the self-excitation current $\overline{{I}_{w}}$ relaxes to zero and this toggles the state back to the quiescent one. Finally, for completeness, at *t* = 3.5 s we apply another distractor perturbation to the resting state to demonstrate its stability too. We note that $\overline{{I}_{w}}(t)$ and *f*(*t*) track each other, which reflects the linearity between these two quantities, as was discussed before.

In Fig. 7 we also apply a ramp-like protocol (center and right panels) to demonstrate the hysteresis effect, as we did in the mathematical model in previous sections. Again, we find the measured data in excellent qualitative agreement with the eQIF model results shown in Fig. 2.

Besides simplicity, an additional attractive feature of our hardware model is that it only uses conventional electronic components, which are affordable and widely available. Thus, this can be of relevance for robotics and control systems based on spiking neural networks. We should mention that the memristive spiking neuron can also be implemented in VLSI^46^, in the case that a massive number of units would be need.

### Networks of self-excitatory neurons and mean-field models

In this section, we return to the mathematical eQIF model to examine the implications of incorporating self-excitatory neurons into network architectures. In this case, the parameter space increases significantly and multiple questions emerge. In this initial analysis, we restrict attention to the most basic issues concerning stability and function.

A first question is whether local self-excitation and persistence might preclude the formation of stable networks composed of such neurons. To address this, we consider the most unfavorable case, namely a fully connected network with purely excitatory synapses—that is, a self-excitatory population of self-excitatory neurons. This is a relevant question, since it is commonly assumed that an excitatory population needs to be partially balanced by an inhibitory one so to avoid runaway rates^11,47^.

If stability is achieved for this kind of network, then a second question to ask is whether the working memory function continues to exist at the population level.

We consider a network of N neurons described by the eQIF model that are globally connected with a probability of connection *P*_*c*_ = 1. The neurons are coupled via the following synaptic current:

19 $${I}_{{{{\rm{syn}}}},i}={g}_{{{{\rm{syn}}}},i}(t)({E}_{{{{\rm{syn}}}}}-{V}_{i}),$$

where, *g*_*syn*,*i*_(*t*) denotes the post-synaptic conductance of neuron i in the network, and *E*_*syn*_ the synaptic reversal potential, i.e. the resting potential of the net synaptic current. In this study, we set *E*_*syn*_ = 0 mV to represent excitatory synapses. The post-synaptic conductance is modeled as a decaying exponential function with an instantaneous increase *w*_*e*_ = 4nS when a pre-synaptic neuron j emits a *k*th spike. Since we consider a network with all-to-all connectivity, each post-synaptic neuron receives the summed input of all the pre-synaptic ones, thus *g*_*syn*,*i*_ = *g*_*syn*_ is homogeneous within the population and we rescale the efficacy by 1/N. Hence the evolution of the post-synaptic conductance is represented by the following differential equation:

20 $$\frac{d{g}_{syn}}{dt}=-\frac{{g}_{syn}}{{\tau }_{syn}}+\frac{{w}_{e}}{N}{\sum }_{j=1}^{N}{\sum }_{{t}_{j}^{k} < t}\delta \left(t-{t}_{j}^{k}\right)$$

By combining the equations for the evolution of the post-synaptic conductance (20) together with that of the single neuron model (7), we obtain the following set of equations for the network of eQIF neurons:

21 $$C\frac{d{V}_{i}}{dt}=	 {g}_{L}({E}_{L}-{V}_{i})({V}_{T}-{V}_{i})+{I}_{w,i}+{I}_{ext}+{g}_{syn}({E}_{e}-{V}_{i})+{\eta }_{i}\\ {\tau }_{w}\frac{d{I}_{w,i}}{dt}=	 a({V}_{i}-{E}_{L})-{I}_{w,i}\\ \frac{d{g}_{syn}}{dt}=	 -\frac{{g}_{syn}}{{\tau }_{syn}}+\frac{{w}_{e}}{N}{\sum }_{j=1}^{N}\sum\limits_{{t}_{j}^{k} < t}\delta \left(t-{t}_{j}^{k}\right)$$

Note that we have included a small quenched intrinsic current term *η*_*i*_ to introduce heterogeneity and avoid trivial synchrony. For later convenience, the heterogeneity term is drawn, for each individual neuron, from a Lorentzian distribution ${{{\mathcal{L}}}}(\eta )$. In our case, we choose for the centroid and the half-width respectively $\bar{\eta }=10$ pA and *Δ*_*η*_ = 0.5 pA.

**Fig. 8:**
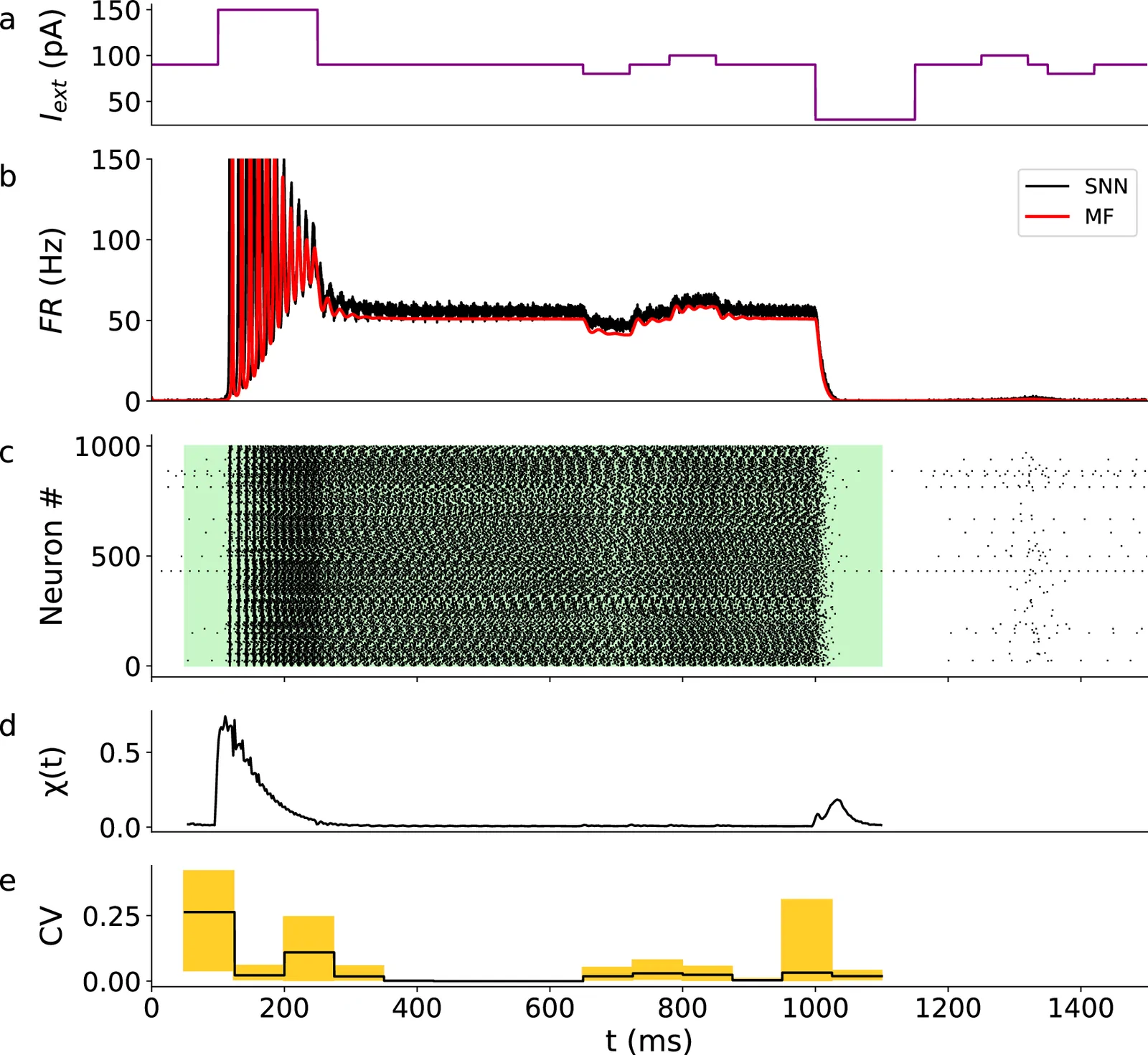
Results of numerical simulation for a network of eQIF neurons (*N* = 5000). Parameter values are reported in Supplementary Information. Neuron state variables $({V}_{i}^{0},{I}_{w,i}^{0})$ are initialized randomly. **a** The network is subjected to an external input protocol, analogous to that applied in the single-neuron simulations described above. **b** Population firing rate (black). The network exhibits both persistent activity and silent states. This panel also shows that the mean-field approximation (red) accurately captures the population firing rate behavior. **c** Raster plot of spiking activity across 1000 randomly selected neurons. The green shaded region indicates the time window used to compute the metrics shown below. **d** Membrane-potential-based synchrony measure, highlighting the increase in synchronization during the stimulus pulse. A slight increase of synchrony is also observed toward the end of the shaded region. This is due to the simultaneous decaying of the membrane potentials across the network. **e** Mean interspike-interval coefficient of variation (CV), computed over sliding windows of 75 ms. Error bars denote the dispersion across the neuronal population.

In Fig. 8 we show simulation results of a large network with *N* = 5000 neurons. We adopt an external current protocol qualitatively similar to the single neuron studies, so to probe the stability and the spiking persistence, i.e. working memory function. Network simulations are realized with the Python package Brian2^48^.

We characterized the reciprocal state of the simulated network using two complementary metrics. Both measures were evaluated only in parameter regions where the mean firing activity was sustained (green shaded area in Fig. 8c), since outside this regime most neurons were silent.

Spike-timing variability was quantified using the coefficient of variation (CV) of the inter-spike intervals (ISIs). For neuron *i*, this is defined as

22 $$C{V}_{i}=\frac{{\sigma }_{{{{{\rm{ISI}}}}}_{i}}}{{\mu }_{{{{{\rm{ISI}}}}}_{i}}},$$

where ${\mu }_{{{{{\rm{ISI}}}}}_{i}}$ and ${\sigma }_{{{{{\rm{ISI}}}}}_{i}}$ denote the mean and standard deviation of its ISIs, respectively. The network-level CV was obtained by averaging *CV*_*i*_ across all neurons. This metric reflects firing regularity, with values close to zero indicating highly regular spiking and values near or exceeding unity corresponding to increasingly irregular or Poisson-like activity.

In addition, network synchrony was assessed via a membrane-potential based synchrony index computed over sliding time windows to capture its temporal evolution. Denoting by $V(t)=\mathop{\sum }_{i=1}^{N}{V}_{i}(t)/N$ the population-averaged membrane potential, synchrony was quantified as

23 $$\chi {(t)}^{2}=\frac{{\sigma }_{V}^{2}}{N\mathop{\sum }_{i=1}^{N}{\sigma }_{i}^{2}},$$

where ${\sigma }_{V}^{2}$ is the variance of *V*(*t*) and ${\sigma }_{i}^{2}$ is the variance of *V*_*i*_(*t*).

Initially, in the subthreshold regime (*I*_*ext*_ = 90 pA), there are only few sporadic spikes, coming from the neurons with higher values of heterogeneity term *η*_*i*_.

At *t* = 100 ms, a strong external pulse is applied, driving the network into a globally excited state. This transition is accompanied by a rapid increase in synchrony, as quantified by *χ*(*t*), which reaches values of ~0.8 near the time of stimulation (see Fig. 8d). At the same time, the CV exhibits a mean value that is rather low ~0.25, but with large dispersion across neurons (see Fig. 8e).

The applied current *I*_*ext*_ is subsequently returned to the subthreshold level at *t* = 250 ms, and we observe that nevertheless the network remains in a persistent spiking state. Persistent activity is also expressed homogeneously at the single-neuron level, as indicated by the CV, which drops to values close to zero with minimal dispersion. This reflects regular firing across neurons, despite the absence of synchrony (*χ*(*t*) ~ 0).

Distractor pulses are applied during the interval (650–850) ms. Although these perturbations modulate the instantaneous spiking response, the overall firing state remains stable.

Hence, these numerical results demonstrate that both stability and working memory function are preserved, even in the limiting case of a full excitatory connectivity network.

Many interesting further questions can be explored in a network model, such as the effect of larger disorder, the possibility of multi-stability, the partition of the population in sub-populations and their interaction and synchrony, etc. They are outside the scope of the present initial study and will be reported elsewhere.

We shall consider one last point, namely the derivation of a mean field model (MFM). This is important, since MFMs are useful tools as they allow to go beyond the scale of large networks, such as in so called whole-brain simulation models^31^. Hence, to that end it is necessary to first formulate and then validate the dynamics of MFMs with numerical simulations against the spiking network. The behavior of the spiking neural network can be characterized in ensemble quantities, such as the population mean firing-rate (see Fig. 8b), that can be compared to the MFM^34,49,50^.

We follow the mean-field derivation originally proposed by Montbrio et al.^34^ for QIF neurons and later expanded by Chen and Campbell to include spike-frequency adaptation (i.e. self-inhibition)^42^. This approach consists in describing the collective dynamics of a population of all-to-all connected heterogeneous neurons in the thermodynamic limit, by considering the evolution of macroscopic observables averaged over the whole network.

To obtain a closed form for the final system of equations it is assumed that neuron’s membrane potential within the population are distributed according to a Lorentzian distribution at all times, with *V*_*peak*_ = − *V*_*reset*_ → *∞*. Under these assumptions, the final mean-field system of equations reads:

24 $$C\frac{dr}{dt} 	={g}_{L}r(2V-{V}_{T}-{E}_{L})-r{g}_{syn}+\frac{{\Delta }_{\eta }{g}_{L}}{\pi C}\\ C\frac{dV}{dt} 	={g}_{L}(V-{V}_{T})(V-{E}_{L})+{I}_{w}+\bar{\eta }+{I}^{*}+{g}_{syn}({E}_{e}-{V}_{T})-\frac{{\pi }^{2}{r}^{2}}{{g}_{L}}\\ {\tau }_{{I}_{w}}\frac{d{I}_{w}}{dt} 	=a(V-{E}_{L})-{I}_{w}+{\tau }_{{I}_{w}}br\\ \frac{d{g}_{syn}}{dt} 	=-\frac{{g}_{syn}}{{\tau }_{syn}}+{w}_{e}r$$

Here *r* denotes the mean firing rate of the network, while all remaining variables represent averages of the corresponding state variables over the network.

To account for finite spike peak and reset potentials, we adopt a correction proposed by Gast et al.^51^. In particular, this is implemented by considering an adjusted current term I^*^, which also includes the external current *I*_*ext*_ (see details in Supplementary Information). This correction partially compensates the discrepancy between the network of neurons and the mean-field reduction, when both *V*_*peak*_ and *V*_*reset*_ assume biological plausible values. The details of the final neural mass model derivation are presented in Supplementary Information.

The behavior of the system of Eq. (24) was obtained numerically and the results are shown in Fig. 8b. As we can observe, the MFM captures in great detail the behavior of the network, including the decay of the synchronicity and the working memory function.

Beyond the proof-of-concept simulations presented here, the relevance of the proposed framework for large-scale applications should be considered in relation to canonical models of working memory and seizure dynamics. Large-scale implementations have often relied on mean-field or neural mass reductions to achieve tractable simulations at cortical or whole-brain scales^34,50^. Similarly, seizure modeling has benefited from dynamical systems approaches, such as neural field or neural mass frameworks, where pathological transitions are interpreted as bifurcations between distinct dynamical regimes^52–54^. From a dynamical systems perspective, both working memory maintenance and epileptic seizures can be viewed as transitions between metastable or attractor states within recurrent circuits; however, they differ in their dynamical signatures. Working memory typically corresponds to stable persistent firing states characterized by moderate, sustained activity, whereas seizures involve transitions toward regimes of abnormally elevated firing rates, increased neuronal synchrony and reduced dynamical variability, often associated with large-scale recruitment of neuronal populations. Existing large-scale models frequently achieve scalability by sacrificing detailed spiking dynamics or simplifying intrinsic mechanisms underlying persistence and excitability. In contrast, the present minimal formulation preserves an explicit spiking description while remaining compatible with analytical mean-field reductions, thereby offering a bridge between microscopic neuron dynamics and macroscopic population models.

## Discussion

In this work, we have introduced a minimal model of a dynamical (or working) memory based on an intrinsic slow self-excitatory current. Our neuron model is a simple modification of highly popular models of computational neuroscience, AdEx, Izhikevich and aQIF, where the self-inhibition (adaptation) is changed into self-excitation by just flipping a sign. The consequence for the behavior of those models is nevertheless significant, they now show a large bi-stability region, without the need of fine tuning, where they exhibit a robust working memory cognitive function.

Besides their numerical efficiency minimal phenomenological models are also relevant because their simplicity reveals with conceptual clarity the key features of the dynamical mechanism that supports the cognitive function. These allowed us to demonstrate that the mechanism can also be exploited to implement a working memory in analog hardware. The excitability of the electronic neuron is realized by the adoption of a memristor device, which can be viewed as a minimal embodiment of a voltage-controlled conductance channel. The circuit is built out of a very small number of widely available and affordable electronic components. We analyzed the persistent spiking state of the circuit in terms of a self-consistent problem, that we solved by means of a geometrical construction. This solution parallels the method used for rate networks, which brings additional intuition to the understanding of the self-sustained spiking state. We have also presented a more formal analysis of the dynamical behavior by means of the nullcline construction of the theoretical model.

Phenomenological models are useful because they facilitate the description of neural dynamics across scales, from single neurons to populations, to networks of populations. We have explicitly demonstrated that our model allows to implement stable networks of densely connected, self-excitatory neurons that are coupled solely with excitatory dynamical synapses. Our results show robust persistent spiking without a synchronization instability, in the absence of any inhibitory coupling, contrary to naive expectations.

We have also derived and numerically validated a mean field model that describes the behavior of the network at the population level. This also opens the way to implement networks-of-networks of excitatory neurons, which may be of interest for whole-brain modeling of epilepsy^52,55–59^.

An exciting feature of our work is that the behavior of our hardware neuron model follows closely the computational neuroscience models that we have considered, both for the single neuron and the mean field. This close association between theory and neuromorphic circuits may open the way for applications of minimal spiking hardware in artificial intelligent systems, robotics and edge medical devices.

## Supplementary information

Supplementary Information

Transparent Peer Review file

## Data Availability

The data associated to this article are available as code and algorithms, with the link provided in the following “Code availability” section.

## Code availability

The code is available on the Ocean Code Project associated to this article and on GitHub at: https://github.com/GabrieleCasagrande/MinimalNeuro_WorkingMemo.
