# Supplementary material for: A minimal model of working memory in neural systems and neuromorphic circuits
Source: Nat Commun. 2026 Aug 17;17:9867. doi: 10.1038/s41467-026-75704-3 (PMC13578268; doi:10.1038/s41467-026-75704-3)
Supplement: Supplementary file 1 [file 41467_2026_75704_MOESM1_ESM.pdf]

SUPPLEMENTARY INFORMATION
A MINIMAL MODEL OF WORKING MEMORY IN NEURAL SYSTEMS AND NEUROMORPHIC CIRCUITS

A. Effect of b term

In this section, we examine the effect of the after-spike jump parameter b (defined in Eq. 9) on the dynamical behavior of the eQIF single-neuron model (Eqs. 7-9).
Supplementary Figure 1a illustrates the wide range of output spiking frequencies generated by the model for different values of b, at fixed values of $I_{\rm ext}$. In particular, increasing the after-spike jump amplitude at constant external input leads to an increase in the average firing frequency. This occurs because larger b values shift the trajectory further away from the attractive basin of the stable fixed point, facilitating sustained spiking activity.
Moreover, we observe that the parameter region supporting bistability expands for larger values of b. For instance, in we focus on the case $b = 50$ pA (Supplementary Fig. 1b), the bistable region is smaller than the one presented in Fig. 2c (corresponding to $b = 100$ pA), reflecting differences in the remaining parameter settings. Nevertheless, the existence of a bistable regime is robust across a broad range of parameter combinations, indicating that bistability does not rely on fine tuning.

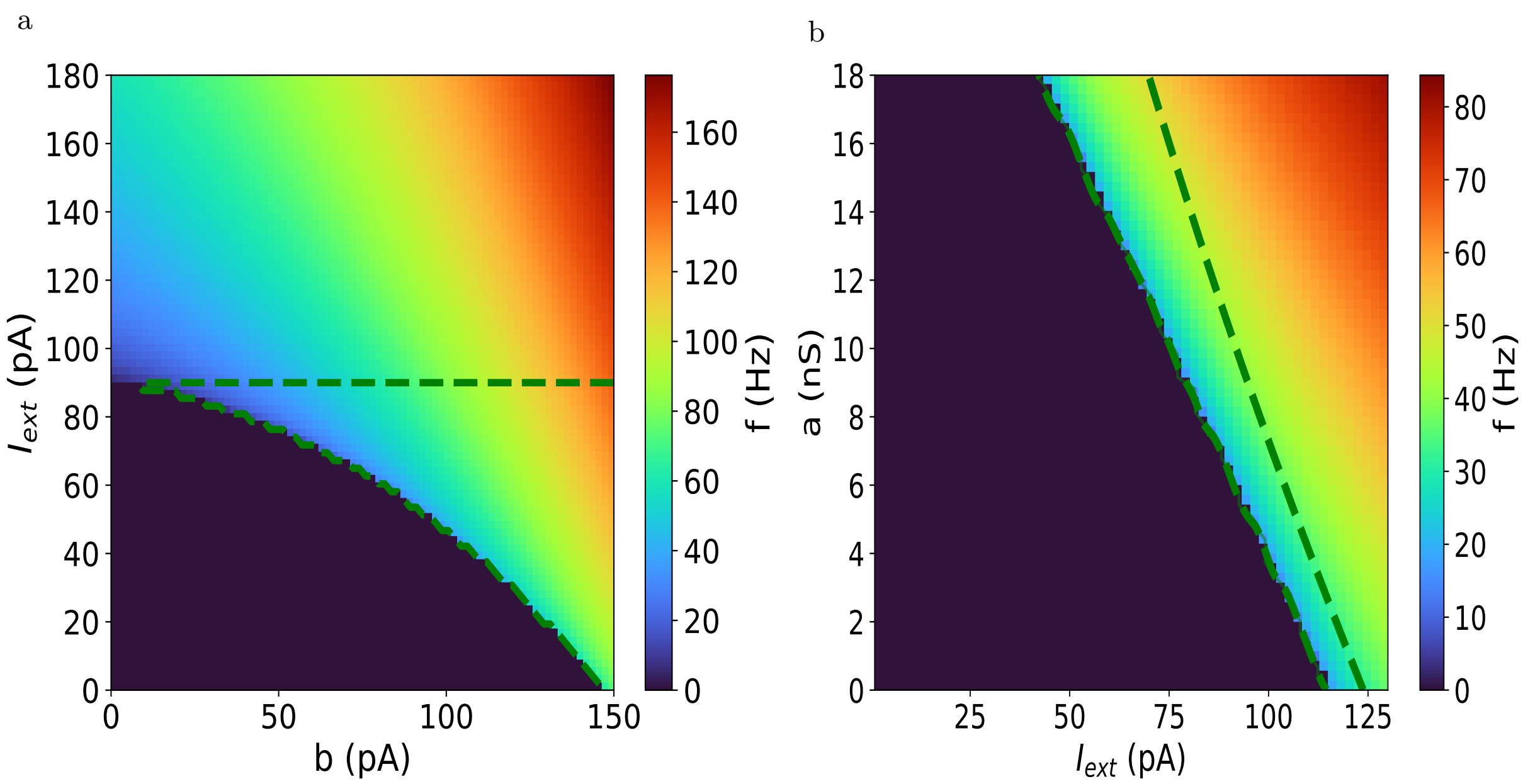

Supplementary Figure 1: Effect of variations in the after-spike jump parameter b on the dynamical behavior of the eQIF single-neuron model. (**a**) Output firing frequency as a function of b, evaluated at fixed levels of external current $I_{\rm ext}$. Hysteresis region is highlighted by dotted green lines. We may observe how for fixed value of the parameter a=10 nS, the hysteresis region is larger as parameter b increases. (**b**) Hysteresis region (bounded by green dotted lines) in the $(I_{\rm ext}, a)$ parameter plane for $b = 50$pA. The heatmap indicates the corresponding output firing frequency. The values of the remaining parameters are the same than those in 1.

B. Stability of single neuron eQIF against perturbations

In this section, we investigate the dynamical stability of the persistent (spiking) and quiescent (resting) states within the hysteresis loop in response to external perturbations. Using the parameter set as in Fig. 1b, we initialize the system in either the persistent or quiescent state. To avoid initial transients effect, we simulate the activity for an initial period of 800ms. Then, we apply a step-current perturbation of varying amplitude and duration to the system. After the perturbation, we continue the simulation for at least an additional 500ms. The final state is classified based on the mean instantaneous firing rate calculated over the last 100ms. A non-zero firing rate identifies a persistent spiking state, while a zero firing rate indicates the quiescent state.

Perturbation amplitudes were negative for the persistent state to induce silencing and positive for the quiescent state to induce activation. We also examined the influence of stochasticity by adding a Gaussian white noise term, $\delta I(t)$, to the input current. This noise term has zero mean, $\langle \delta I(t) \rangle = 0$pA, and variable standard deviation σ_{noise}. The baseline current was fixed at $I_{base} = 65$pA, positioned at the center of the hysteresis loop shown in Fig. 2b. Representative traces of these perturbed input currents are illustrated in Supplementary Fig. 4.

Initially, we evaluated the stability of both states under deterministic (noiseless) perturbations. The results, summarized in Supplementary Fig. 2, demonstrate that for low perturbation amplitudes (specifically $\lesssim 25$pA), the system remains within its original hysteresis branch, and both states remain stable regardless of the perturbation duration. As the amplitude increases, the minimum duration required to trigger a state transition decreases. Nevertheless, even at high perturbation amplitudes (~ 90pA), both states exhibit robustness against very brief perturbations ($\lesssim 10$ms). Conversely, for amplitudes only slightly exceeding the branch stability thresholds (~ 25pA), the state remains stable unless the perturbation is sustained over a significantly long period.

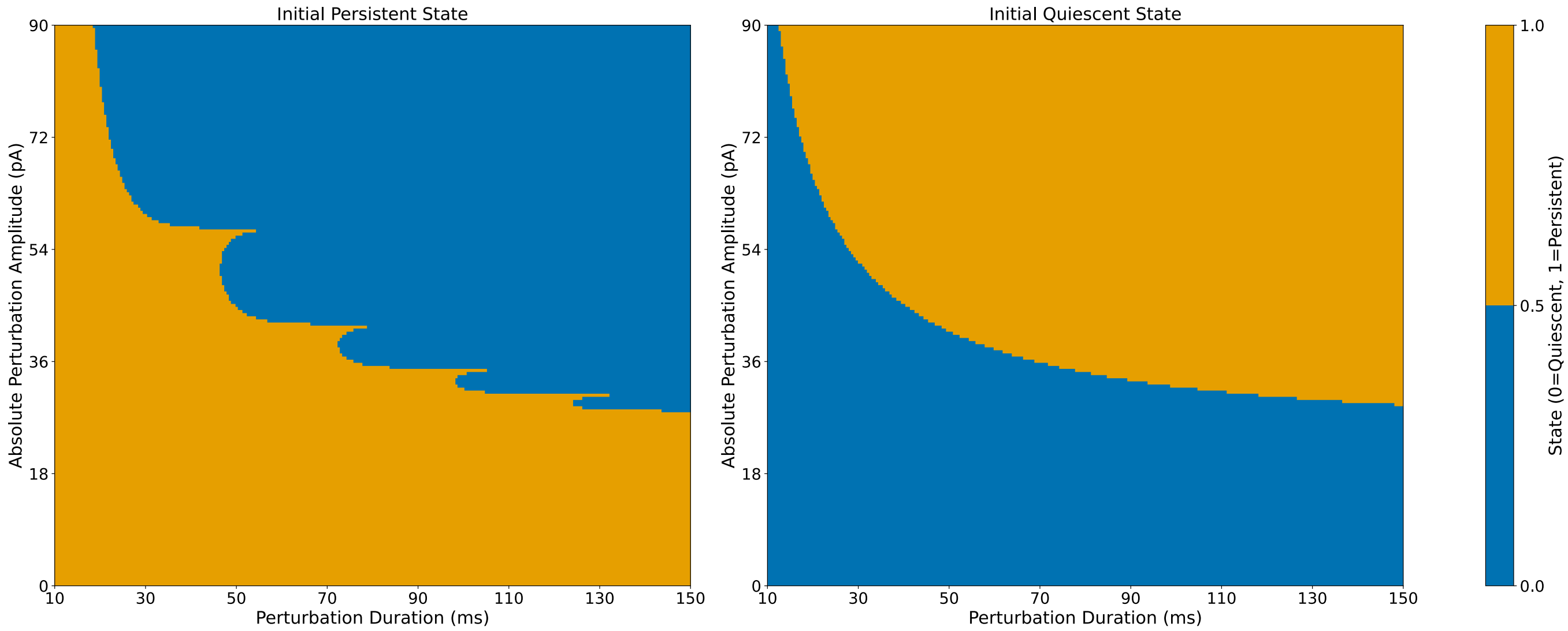

Supplementary Figure 2: Effect of perturbation amplitude and duration on the stability of persistent and quiescent states within the hysteresis region. The left and right heatmaps represent the stability of the persistent spiking and quiescent states, respectively. Given an initial baseline current at the center of the hysteresis loop (Fig. 2b), the states remain stable for perturbation amplitudes below the branch threshold (~ 25pA) across all durations. The perturbation applied is negative for the initial persistent spiking stae (left) and positive for the quiescent one (right). Beyond this threshold higher amplitudes require shorter durations to induce a transition. In the persistent state (left), a structured pattern appears in the transition boundary, arising from the phase-dependent interplay between the spiking frequency and the perturbation timing.

Next, we assessed the impact of additive noise on state stability. We repeated the previous analysis while systematically increasing the noise intensity σ_{noise}. Supplementary Fig. 3 displays the resulting transition boundaries for different noise levels. These switching lines represent the couples of amplitude and duration values of the perturbation which result into a transition from the initial state to the other one. Our results indicate that noise does not significantly compromise the stability of either state. Specifically, increasing σ_{noise} results in negligible shifts in the transition boundaries. Since the noise has a zero mean ($\langle \delta I(t) \rangle = 0$), its fluctuations are effectively integrated out and do not alter the deterministic switching thresholds.

To visualize the single-neuron dynamics under these conditions, we plotted the instantaneous firing rate in response to a 100ms perturbation across various noise intensities (Supplementary Fig. 4). For clarity, we focus on the persistent spiking state, though the quiescent state exhibits analogous behavior. As shown in Supplementary Fig. 4, even high-intensity noise merely induces small fluctuations in the instantaneous firing rate without shifting the average frequency or triggering an unintended transition.

In conclusion, when initialized at a robust operating point within the hysteresis region, both the persistent and quiescent states of the eQIF neuron demonstrate significant resilience to both transient external perturbations and continuous stochastic noise.

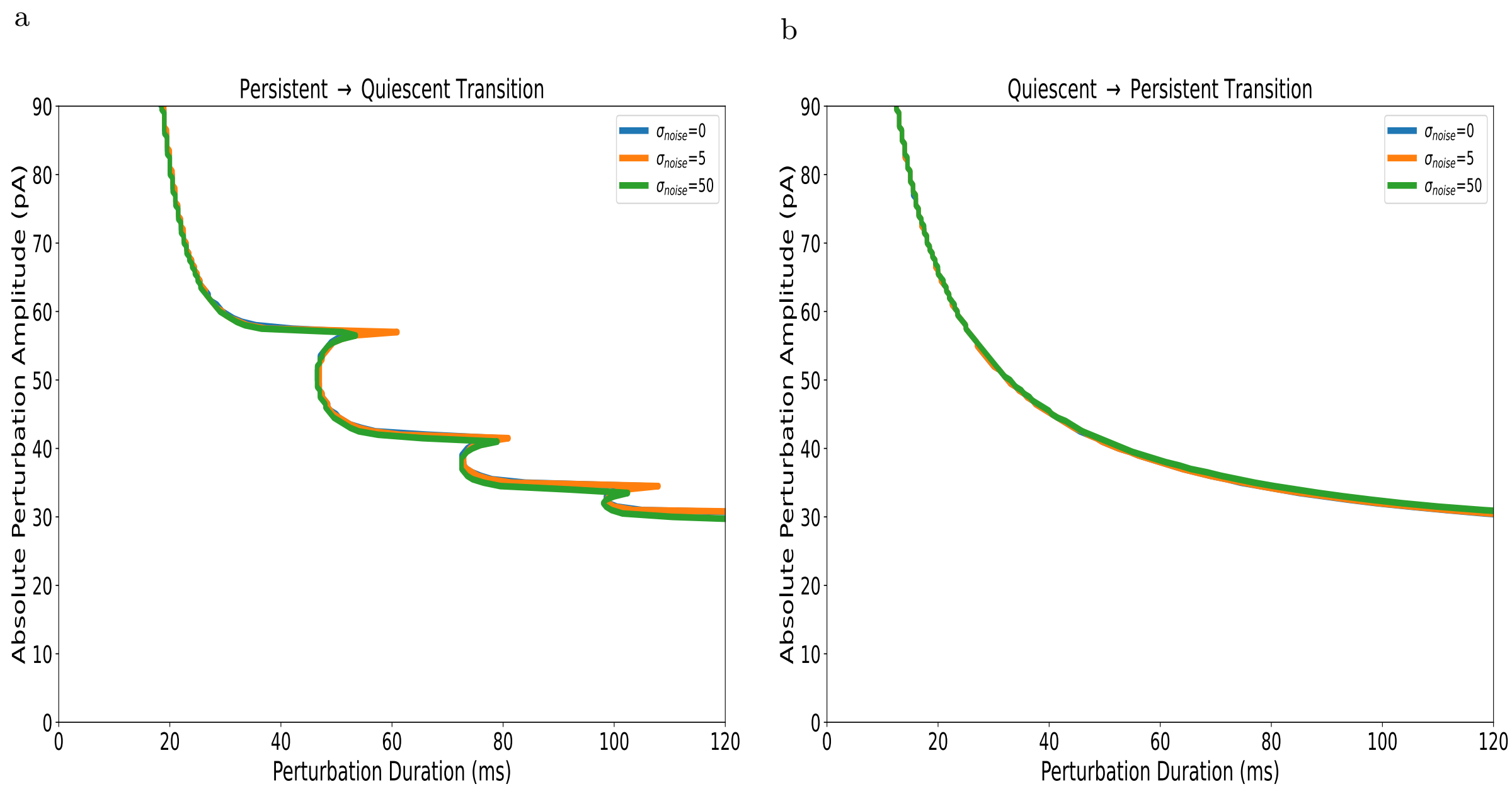

Supplementary Figure 3: Lack of influence of noise on the transition boundaries between states. (**a**) Transition from persistent spiking to quiescence. (**b**) Transition from quiescence to persistent spiking. The transition lines, computed for varying noise intensities (color-coded), show minimal deviation from the deterministic case, suggesting that the bistable states are robust against stochastic fluctuations.

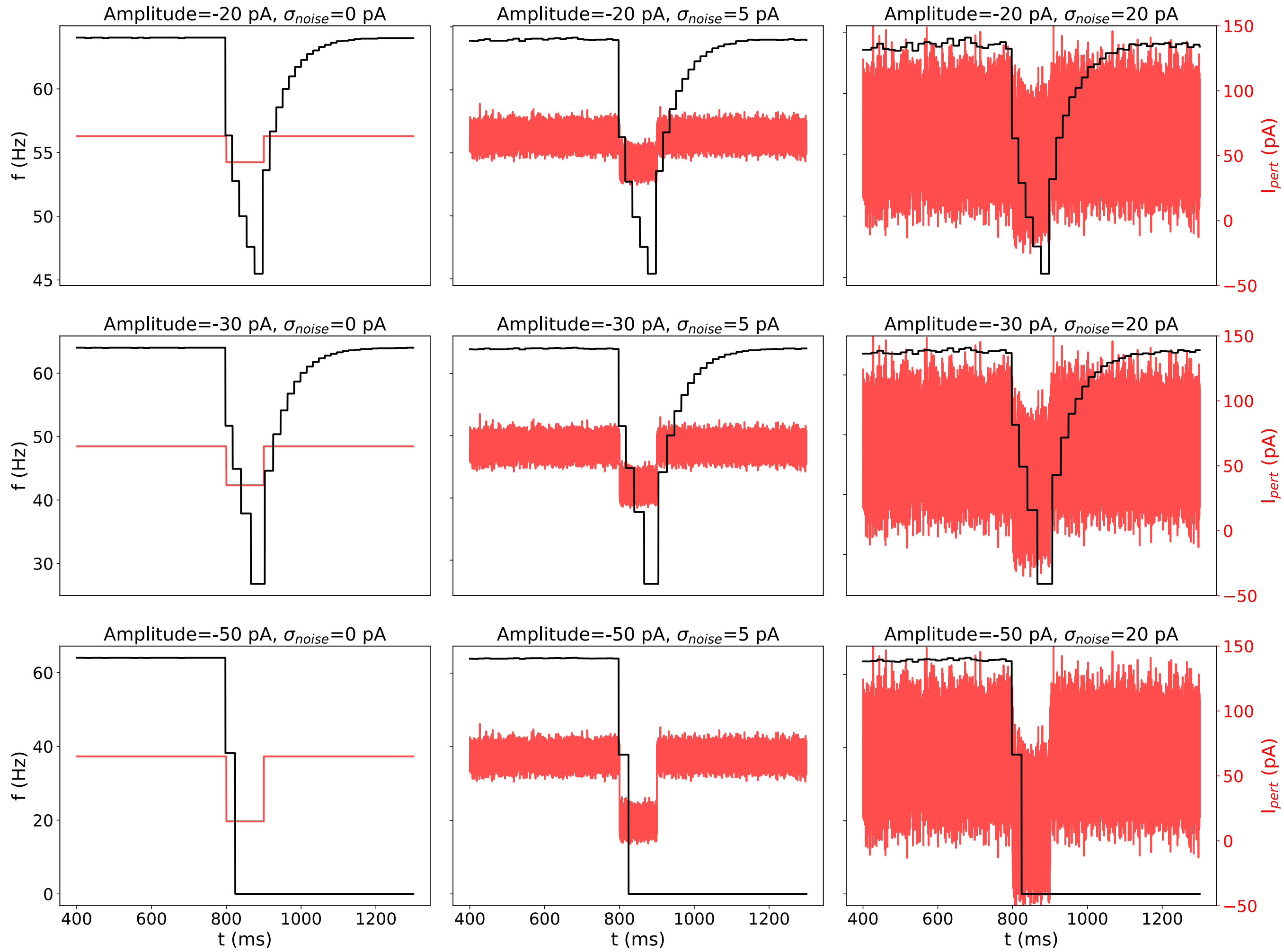

Supplementary Figure 4: Response of the instantaneous firing rate to noise and perturbations. Each panel shows a 1400ms simulation of the eQIF model ($I_{base} = 65$pA) with a 100ms perturbation applied at $t = 800$ms. Rows correspond to increasing perturbation amplitudes. The first column represents the noiseless case, while subsequent columns show increasing noise intensities. The noise introduces fluctuations in the firing rate but does not fundamentally disrupt the stability of the state.

C. Parameters of the eQIF model

This section details the parameter values for the eQIF model used in the corresponding figures. Parameters not explicitly listed here are consistent with the values defined in the main text.
Unless stated otherwise, the simulations for Figures 1 and 2 utilize the parameters listed in Table I. Instead, for Figures 4 and 3 we use the parameters in Table II.

g_L	10 nS/mV	V_T	-55 mV
C	200 pF	E_L	-62 mV
V_{peak}	-20 mV	V_{reset}	-70 mV
a	4 nS	b	100 pA
τ_{I_w}	20 ms		

TABLE I: Parameters values for the eQIF neuron model adopted for Figures 1 and 2.

g_L	10 nS/mV	V_T	-55 mV
C	200 pF	E_L	-65 mV
V_{peak}	-20 mV	V_{reset}	-58 mV
τ_{I_w}	20 ms		

TABLE II: Parameters values for the eQIF neuron model adopted for Figures 3 and 4.

D. Other Models

Similar results can be achieved using other non-linearities. In this appendix we propose two examples, using the exponential integrate-and-fire model and the Izhikevich model. We also show for the eQIF model that similar results can be obtain without dependence on V in the self recurring excitation current equation. Finally, we additionally show that qualitatively similar results can be achieve with a simple Leaky Integrate-and-Fire model with self-excitation.

1. *Exponential integrate-and-fire*

Modifying the original AdEx [28], we obtained the following equations:

$$C\frac{dV}{dt} = g_L(E_L - V) + g_L\Delta_T \exp\left(\frac{V - V_T}{\Delta_T}\right) + I_w + I_{\text{ext}}$$
$$\tau_w\frac{dI_w}{dt} = a\,(V - E_L) - I_w$$

With the reset condition:

$$\text{if} \quad V = V_{spike} \quad \text{then} \quad I_w \leftarrow I_w + b$$

The parameters used for Supplementary Fig. 5 are $C = 180\,\text{pF}$, $g_L = 10\,\text{nS}$, $E_L = -65\,\text{mV}$, $V_T = -55\,\text{mV}$, $\Delta_T = 2\,\text{mV}$, $\tau_w = 20\,\text{ms}$, $a = 2.5\,\text{nS}$, $b = 60\,\text{pA}$, and $V_{\text{reset}} = -58\,\text{mV}$.

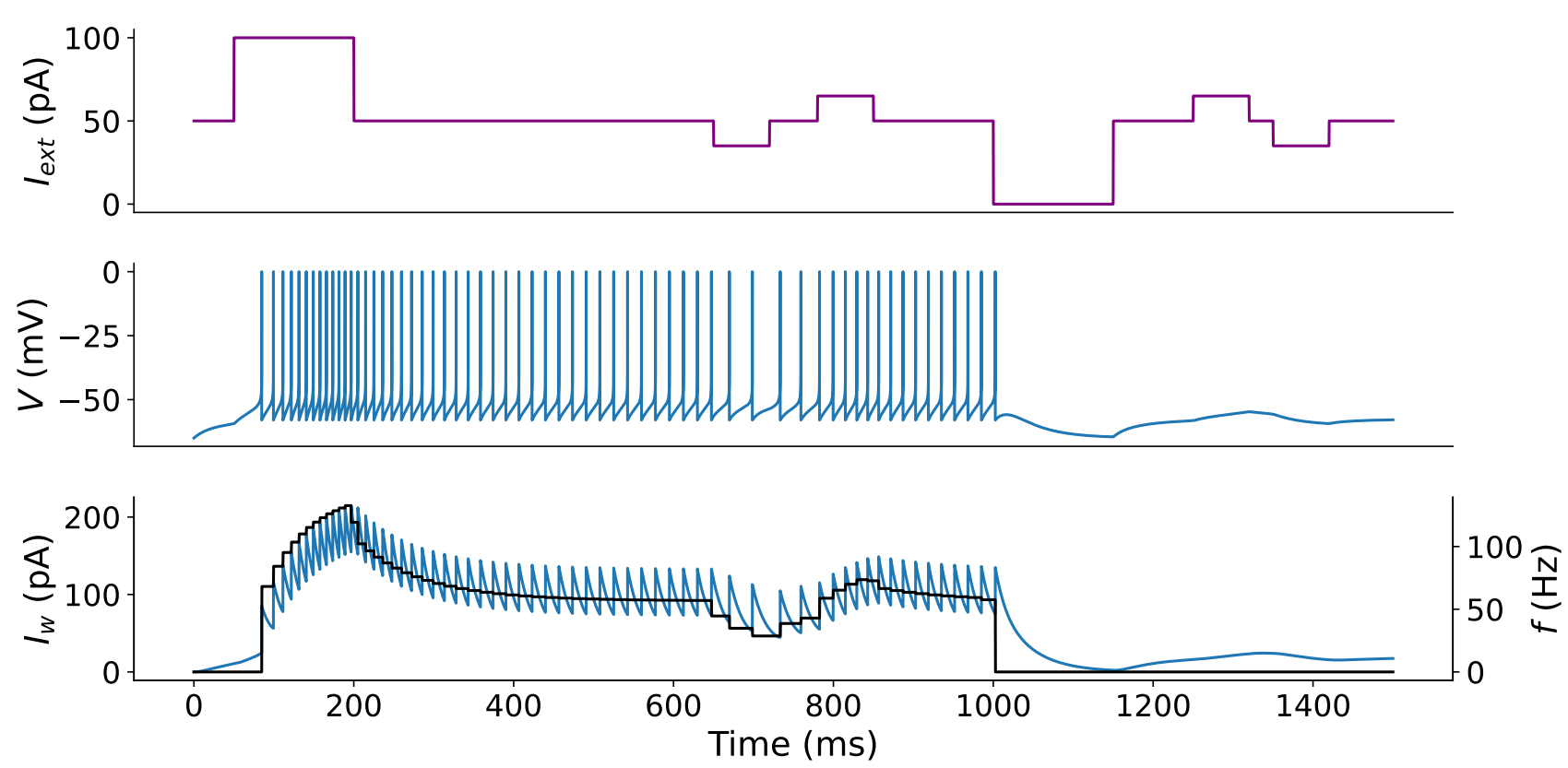

Supplementary Figure 5: Response of the dynamical memory attractor based on the Exponential Integrate-and-Fire model.

2. *Izhikevich model*

Based on the original Izhikevich model [25], modified with a positive I_w, we obtain the following equations:

$$\frac{dV}{dt} = 0.04V^2 + 5V + 140 + I_w + I$$
$$\frac{dI_w}{dt} = a(bV - I_w)$$

With the reset condition:

$$\text{if} \quad V = V_{spike} \quad \text{then} \quad I_w \leftarrow I_w + d$$

We obtained qualitatively similar dynamics as shown in Supplementary Fig. 6.

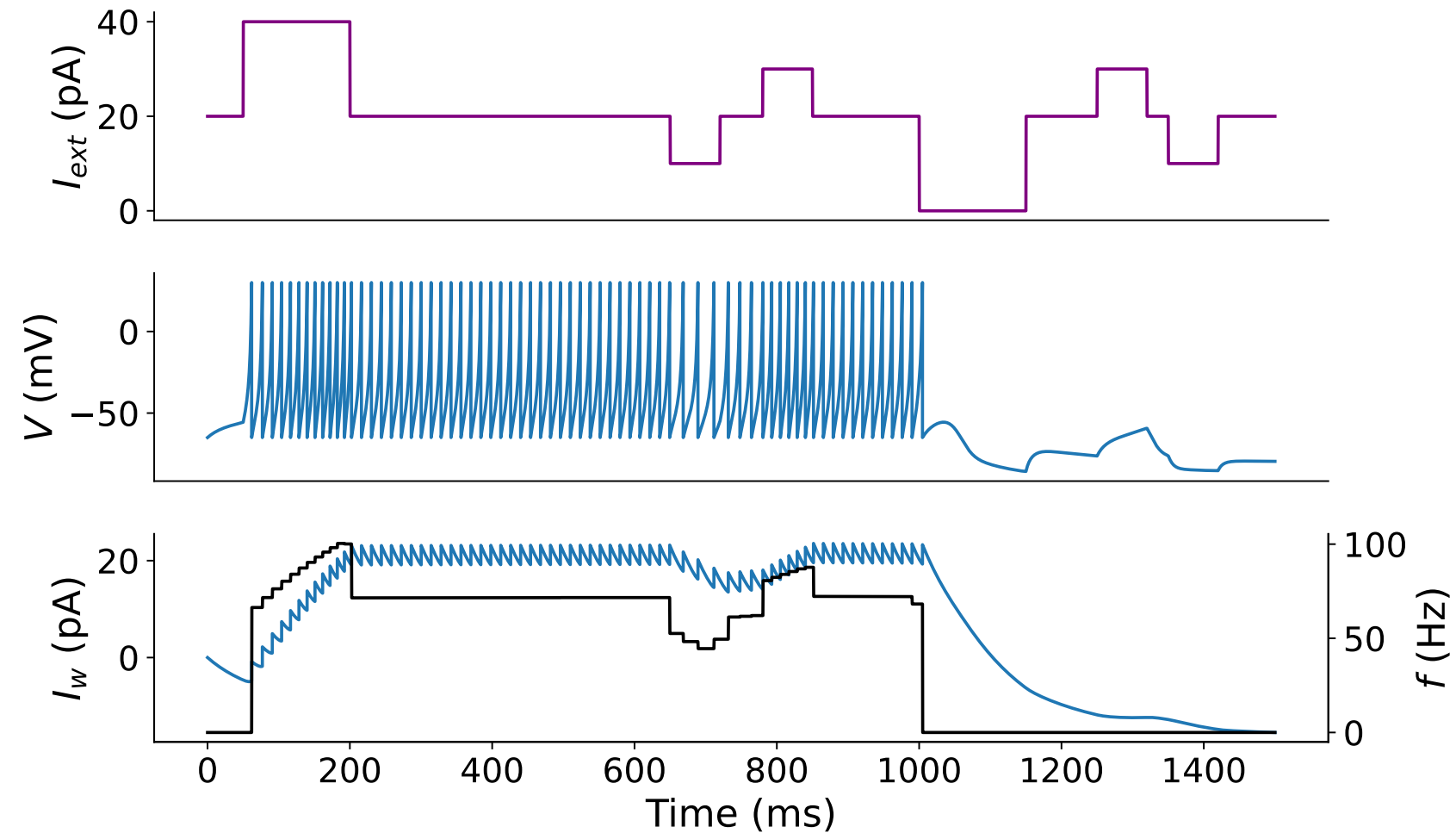

Supplementary Figure 6: Response of the dynamical memory attractor based on the Izhikevich model, with $a = 0.1$, $b = 0.2$ and $d = 4$

3. Quadratic Leaky Integrate-and-Fire

For the eQIF model presented in the main part of this article, we can remove the dependence of the I_w current on V by setting $a = 0$, i.e. $H(V) = 0$ (cf. Eq. 6). This brings the model qualitatively closer to our hardware circuit model. The equation then becomes:

$$C\frac{dV}{dt} = g_L(E_L - V)(V_T - V) + I_w + I_{ext} \tag{25}$$

$$\tau_w \frac{dI_w}{dt} = -I_w \tag{26}$$

$$\text{if} \quad V = V_{spike} \quad \text{then}$$

$$V \leftarrow V_{reset} \quad \text{and} \quad I_w \leftarrow I_w + b \tag{27}$$

Under this condition we maintain the bistability properties as shown in Supplementary Fig. 7

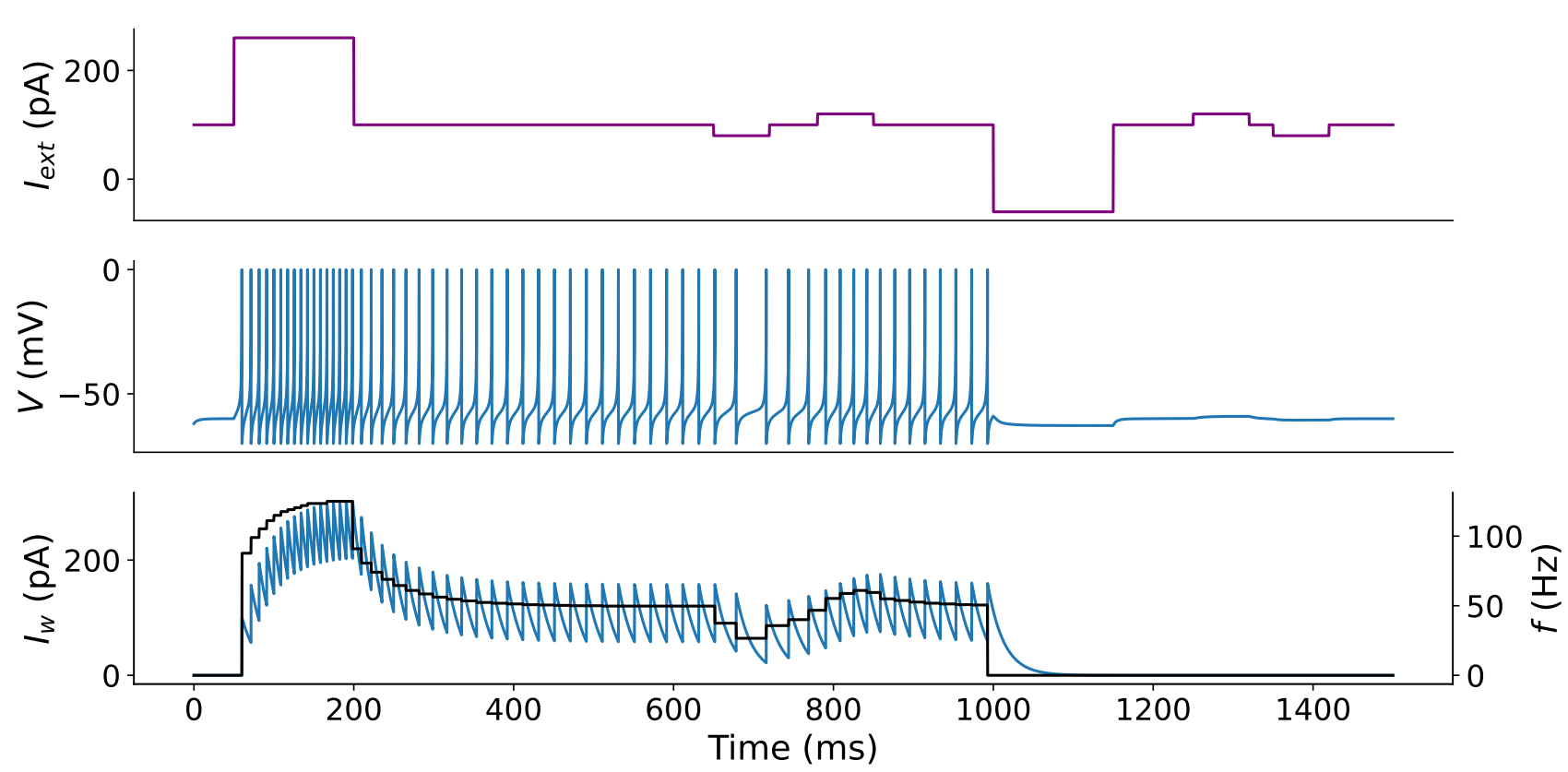

Supplementary Figure 7: Response of the dynamical memory attractor based on the eQIF model without dependence of I_w on V ($a = 0$).

4. Leaky Integrate-and-Fire

The closest model to our hardware circuit is the LIF model with $H(V) = 0$ in the slow variable. Hence, we show here that the standard Leaky Integrate-and-Fire (LIF) supplemented by the simplest version of the slow (positive) self-excitatory current I_w, i.e. $a = 0$ and $b \neq 0$ also shows robust persistence and bistability. The equations read:

$$C\frac{dV}{dt} = g_L(E_L - V) + I_w + I$$
$$\tau_w \frac{dI_w}{dt} = -I_w$$

With the reset condition:

$$\text{if} \quad V = V_{spike} \;\; \text{then} \quad I_w \leftarrow I_w + b$$

We obtained qualitatively similar dynamics as shown in Supplementary Fig. 8.

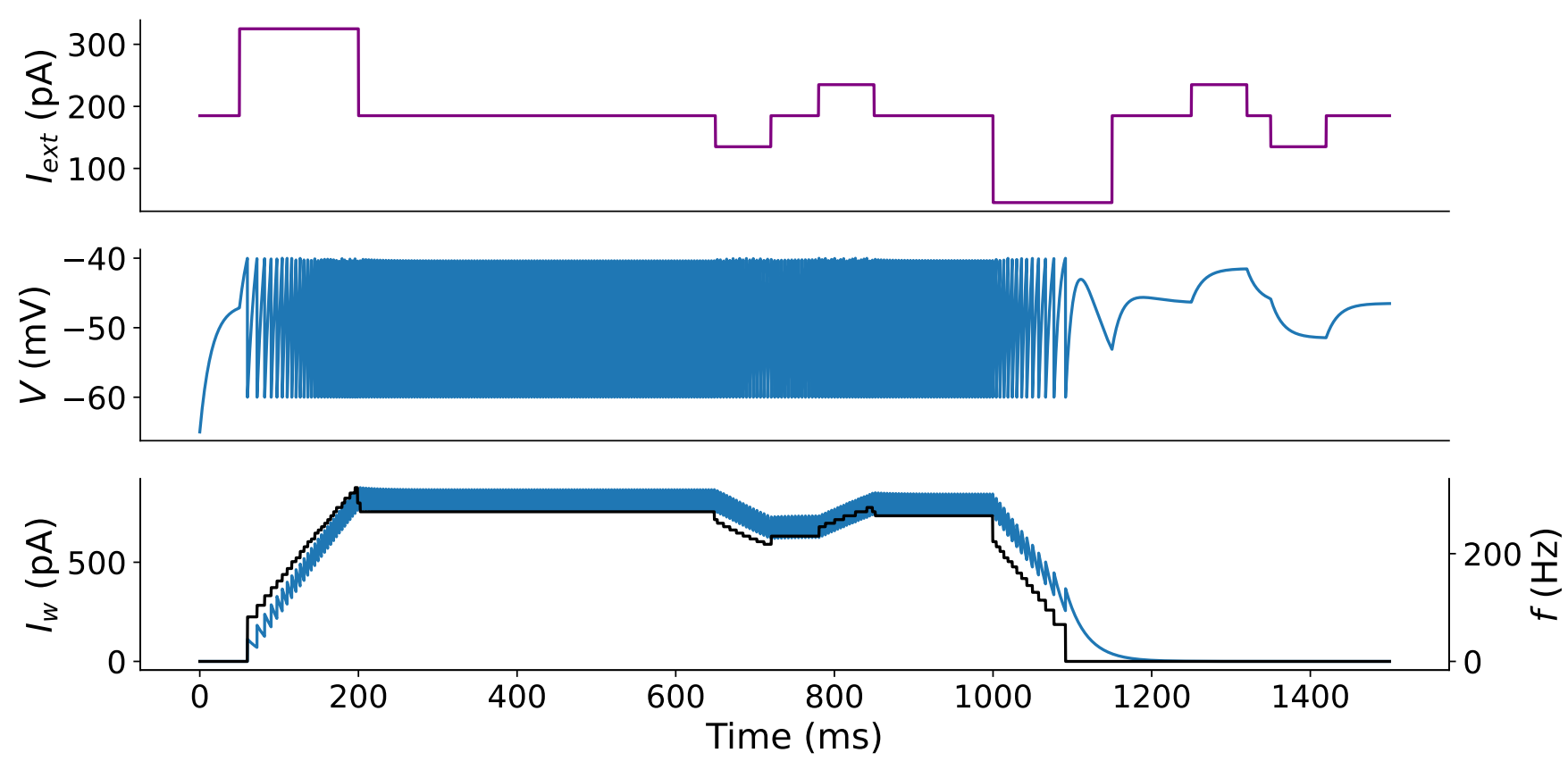

Supplementary Figure 8: Response of the dynamical memory attractor based on the LIF model

E. Mean-field system derivation

In this appendix we present the mathematical steps of the mean-field derivation in the subsection C of the results. In particular, we are interested in computing averaged variables, which are suited to describe a population of spiking neurons:

- Mean membrane potential:

$$V(t) = \int_{\partial\eta}\int_{\partial I_w}\int_{\partial V} V\rho(t, V, I_w, \eta)d\eta dI_w dV \tag{28}$$

- Mean adaptation current:

$$I_w(t) = \int_{\partial\eta}\int_{\partial I_w}\int_{\partial V} I_w\rho(t, V, I_w, \eta)d\eta dI_w dV \tag{29}$$

- Population firing rate, namely the flux of neurons through V_{peak} over the whole range of I_w and η:

$$r(t) = \int_{\partial\eta}\int_{\partial I_w} J^v(t, V_{peak}, I_w, \eta)d\eta dI_w \tag{30}$$

The evolution of the system variables, in the thermodynamical limit, describes a continuous flow in the space of variables. The state of the system is described, at time t, by $\rho(t, V, I_w, \eta)$, which represents the density of neurons

with particular values of the coordinates.
The following continuity equation holds (since the number of neurons is assumed constant in time):

$$\frac{\partial \rho}{\partial t} + \nabla \cdot J(t, V, I_w, \eta) = 0 \tag{31}$$

Where $J(t, V, I_w, \eta)$ is the probability flux, defined as:

$$J = \begin{pmatrix} J^V \\ J^{I_w} \end{pmatrix} = \begin{pmatrix} \frac{1}{C}\left[g_L(E_L - V)(V_T - V) + I_w + I_{syn} + I_0 + \eta\right] \\ a(V - E_L) - I_w \end{pmatrix} \rho(t, V, I_w, \eta) \tag{32}$$

With the reset conditions:

$$J^V(V_{peak}, I_w) = J^V(V_{reset}, b + I_w) \tag{33}$$

A simplified equation for I_w can be derived by consider an adiabatic approximation, which is based on the assumption that the dynamics of individual membrane potential v_i is much faster than that of the adaptation current $I_{w,i}$. This is valid when spike-frequency adaptation is small, meaning $I_w >> b$. Under this assumption, the dynamics of I_w can be approximated by replacing the spike train of the individual neuron with that averaged over the all the neurons within the network:

$$j(t) = \frac{1}{N} \sum_{i=1}^{N} \sum_{t_i^k < t} \delta(t - t_i^k)$$

In the thermodynamical limit $j(t) \to r(t)$ [60], hence we recover the average population firing rate r(t):

$$\dot{I_w}(t) = a(V(t) - E_L) - I_w(t) + \tau_{I_w} b r(t) \tag{34}$$

The averaged spiking activity over the all the neurons within the network $j(t)$, also appears to the post-synaptic conductance variable $g_{syn}(t)$. Therefore, its evolution in time can be related once again to the population firing rate leading to the equation:

$$\frac{dg_{syn}}{dt} = -\frac{g_{syn}}{\tau_{syn}} + w_e r, \tag{35}$$

In order to compute the mean-field equation for the remaining variables we focus on the "reduced" continuity equation for the membrane potential:

$$\frac{\partial}{\partial t} \rho^V(t, V|\eta) = -\frac{1}{C} \frac{\partial}{\partial V} \left[\left(g_L(E_L - V)(V_T - V) + I_w + I_{syn} + I_0 + \eta \right) \rho^V(t, V|\eta) \right] \tag{36}$$

We proceed by making an ansatz for the functional form of $\rho^V(t, V|\eta)$, by noticing that (36) has a Lorentzian shaped stationary solution. Thereby, we assume that it follows a distribution of the same functional form in time [34, 61], leading to:

$$\rho^V(t, V|\eta) = \frac{1}{\pi} \frac{x(t, \eta)}{\left[V - y(t, \eta)\right]^2 + x^2(t, \eta)}, \tag{37}$$

with $x(t, \eta)$ and $y(t, \eta)$ respectively the half-width at half-maximum and the the centroid of the distribution.
In the limit $V_{peak} = -V_{reset} \to \infty$, one can express the interesting average observables in term of the Lorentzian distribution parameters:

$$V(t) = \int_{\partial \eta} y(t, \eta) \mathcal{L}(\eta) d\eta \tag{38}$$

$$r(t) = \int_{\partial \eta} \frac{g_L}{C\pi} x(t, \eta) \mathcal{L}(\eta) d\eta \tag{39}$$

g_L	-20 nS/mV	a	4 nS
C	200 pF	b	20 pF
V_T	-55 mV	τ_{I_w}	20 ms
E_L	-62 mV	w_e	4 nS
E_e	0 mV	τ_{syn}	6 ms
V_{peak}	-20 mV	$\bar{\eta}$	10 pA
V_{reset}	-70 mV	Δ_η	0.5 pA

TABLE III: Parameters values for the eQIF neuron model adopted for the spiking neural network and mean-field

Thus, we proceed to plug (37) into the reduced continuity equation (36) to obtain the time evolution for $x(t,\eta)$ and $y(t,\eta)$.
We obtain a second order equation in v and equating the coefficients of v^2 and v to 0, we find respectively:

$$\dot{x}(t,\eta) = \frac{x\left(2g_L y - E_L g_L - g_L V_T - g_{syn}\right)}{C}$$
$$\dot{y}(t,\eta) = \frac{g_L(y-V_T)(y-E_L) + I_{ext} + I_w + \eta + g_{syn}(E_e - y) - g_L x^2}{C}$$

Both of the results, lead to the disappearance of the coefficient related to v^0 (i.e. the equation holds for each value of v).

To achieve the final set of mean-field equations, we need to specify a functional form for the heterogeneity distribution to evaluate (38) and (39). Once again we assume that $\mathcal{L}(\eta)$ is distributed according to a Lorentzian distribution. This is an arbitrary choice that allows to simplify the computation. Different functional forms are possible, however, as demonstrated by Montbrio et al. [34], results are qualitatively similar. With this assumption we have that:

$$\mathcal{L}(\eta) = \frac{1}{\pi}\frac{\Delta_\eta}{[\eta - \bar{\eta}]^2 + \Delta_\eta^2} \tag{40}$$

with Δ_η and $\bar{\eta}$ respectively the half-width at half-maximum and the centroid of the distribution. Finally, by exploiting the residue theorem we then achieve the final mean-field set of equations (24).

A limiting assumption on the derivation of the mean-field model is the assumption that $V_{peak} = -V_{reset} \rightarrow \infty$. Discrepancies between spiking network and mean-field model due to spike resetting mechanism can be accounted for by introducing an adjusted additive current final equation of the system, as done by Gast et al. [51]. The correction term can be derived by evaluating the impact of V_{peak} and V_{reset} on the spiking dynamics, from single neuron to network dynamics. In order to do so, we exploit the adiabatic approximation on individual adaptation current $I_{w,i}$, which allows to compute a closed analytical solution of $\dot{V}_i$ (8):

$$V(t) = \frac{1}{2}\left[\sqrt{\mu_i} tan\left[\frac{g_L\sqrt{\mu_i}t}{C} + tan^{-1}\left(\frac{2V_{reset} - A/g_L}{\sqrt{\mu_i}}\right)\right] + \frac{A}{g_L}\right] \tag{41}$$

where $\mu_i = \frac{4B}{g_L} - (\frac{A}{g_L})^2$. For the sake of clarity, we identified $A = g_L(V_T + E_L) + g_{syn}$ and $B = I_w + \eta + I_0 + g_{syn}E_e$. We point out that the adjusted current affect just the spiking regime, namely when $\mu_i => 0$. In fact with this choice $V(t)$ blows up, hence the neuron fires a spike. From the solution we can compute the individual neuron firing rate r_i by taking the inverse of the time to reach V_{peak}. It depends on both peak and reset potential values, thus we can than evaluate the mismatch due to the infinite limit. To correct this mismatch we introduce an adjusted current I_i^* at single neuron level. An identical input I^* is added to all neurons, neglecting effects due to single fluctuation of η_i to the correction term. At mean-field level the correction is the following:

$$I^* = \begin{cases} \frac{g_L\mu\pi^2}{4\gamma^2} + \frac{\alpha^2}{4\gamma^2} - g_L E_L v_\theta - g_{syn}E_e - I_w - \bar{\eta}, & \text{when } \mu > 0 \\ I_0, & \text{otherwise} \end{cases} \tag{42}$$

F. Hardware Implementation. Detailed Schematics

As depicted in Supplementary Fig. 9, the circuit is composed by four main units, from left to right: a current source to the neuron, the neuron itself, a spike pulse-current source and the slow self-excitation variable. The neuron

receives as input two currents: an external constant current I_{ext} and a self-excitatory synaptic current $I_w(t)$. Both currents charge the capacitance C_m until the voltage across the capacitance (V_m) reaches a threshold V_{th}. Then the memristor $T + R_m$ switches to high conductance, provoking the discharge of C_m on the output resistance R_a and producing a spike (V_{spike}). The action potential V_{spike} produces a current pulse $B(V)$, which gets filtered on the last unit the slow variable filter.

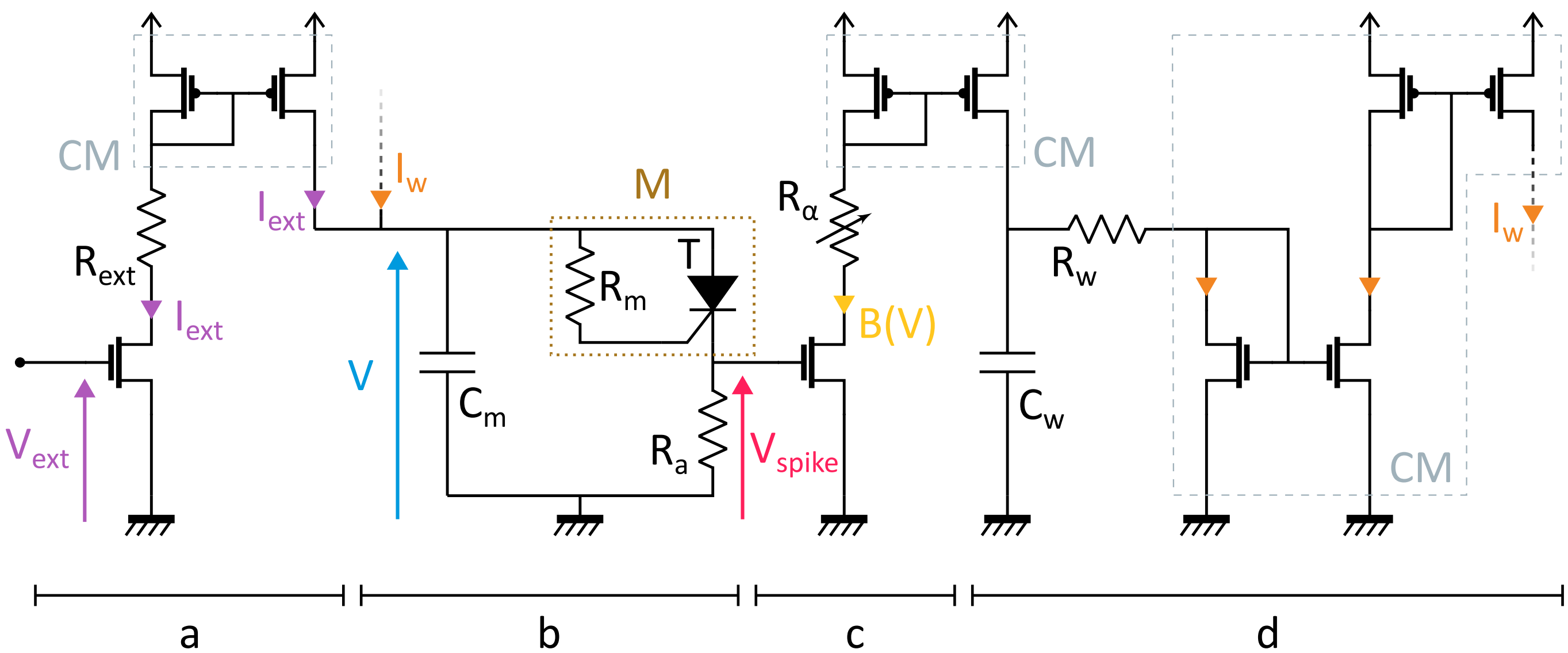

Supplementary Figure 9: Electronic Circuit of a Minimal Working Memory Neuron Model. (**a**) Continuous Current Source I_{ext}. (**b**) Memristive aLIF Neuron. (**c**) Pulse Current Source $B(V)[R_\alpha]$. (**d**) Slow Current Variable I_w. The schematic explicitly shows all the discrete analog components : The thyristor T (P0118MA device), which connected to the resistor R_m (680kΩ) implements the two-terminal memristor device M (golden box). The membrane capacitor C_m (220nF), and the spike output resistor R_a (2.2kΩ). The different transistors (multiple ALD1105PBL) implement the current sources by current-mirror layouts (CM, gray boxes). The current levels ($I_{on} \approx 4V/R$) of I_{ext} and $B(V)$ are tuned by the potentiometers R_{ext} ($\in [0, 250]k\Omega$) and R_α ($\in [0, 100]k\Omega$), respectively. The latter controls the parameter α, which sets the proportionality between f and $\overline{I_w}$ ($\overline{I_w} = f/\alpha$). Finally, R_w (10kΩ) and C_w (2.2μF) set the slow variable time constant τ_w ($R_w Cw = 22ms$). All the components are conventional, affordable and widely available.

$R_{ext} \in$	[0, 250] kΩ	C_m	220 nF	$R_\alpha \in$	[0, 100] kΩ
T	P0118MA	R_m	680 kΩ	R_w	10 kΩ
CM	ALD1105PBL	R_a	2.2 kΩ	C_w	2.2 μF

TABLE IV: Hardware Model Component Values

Supplementary Fig. 10 shows the geometric construction analog to that shown in Fig. 6 for two additional cases of feedback intensity

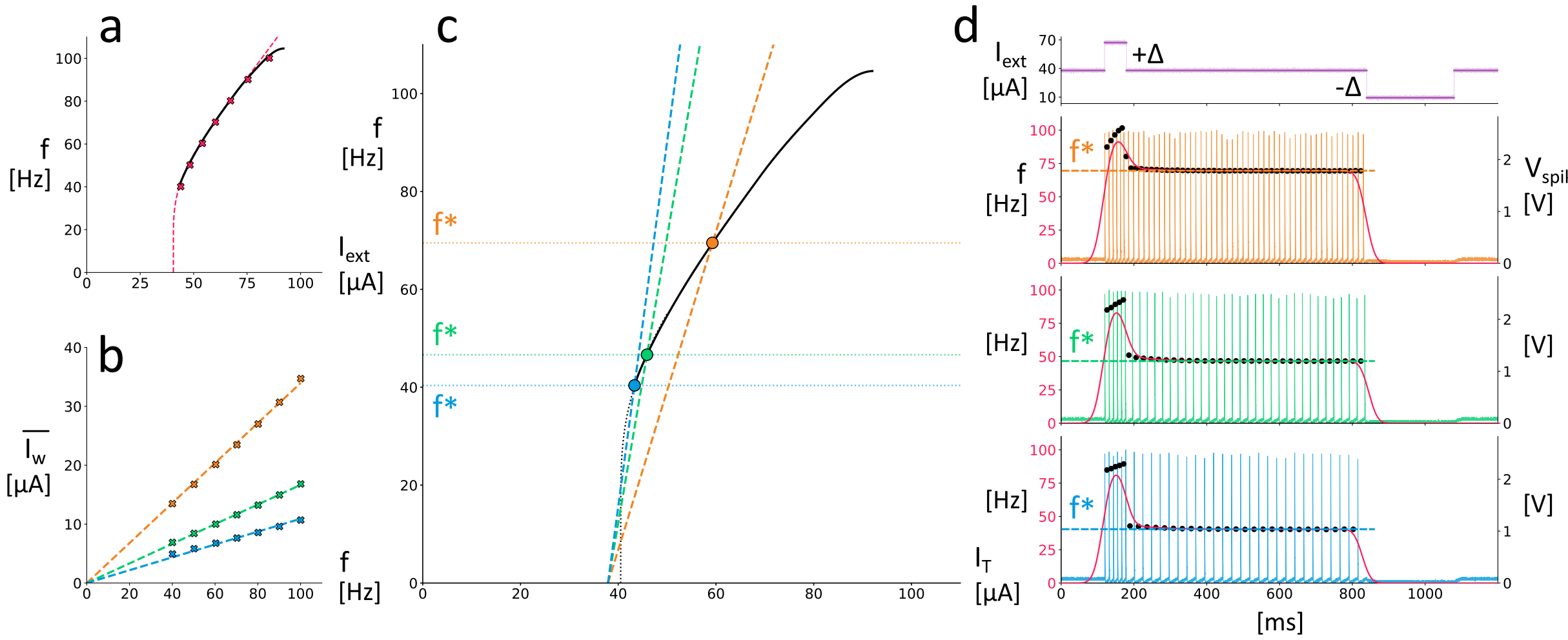

Supplementary Figure 10: Panel **a**: Measured spiking frequency response as function of input current $f(I_{ext})$ in open-loop, i.e. $I_{ext} = I_T$, indicated by red-crosses. In red dashed-line is the fit with the LIF response form $A/[1 - B\ log(1 - I/I_{min})]$ with A and B constants and I_{min} the threshold value for excitation of the input current I_{ext}. Panel **b**: Measured slow current as function of the spiking frequency $\overline{I_w}(f)$ in open-loop, i.e. no feed-back, indicated by orange crosses. From the data $\overline{I_w} \approx (1/\alpha)f$ indicated by dashed-lines. They correspond to three values of the slope α which is controlled by the resistance R_α in the circuit model above. Panel **c**: Geometric construction of the self-consistent solution (I_T^*, f^*) indicated by the three circles with the same color code as in panel **b**. The black line is the response from panel **a**, the dashed-lines are the data of panel **b** (flipped 90 degrees and shifted by I_{ext}). They respectively correspond to the equations 17 and 18 of the main text. Panel **d**: Time dependent traces during the working memory function. (top) Applied protocol of input current $I_{ext}(t)$ with a subthreshold base-line $I_{ext} = 38\mu$A and $\pm\Delta$ excitatory and inhibitory pulses. (below, top to bottom) measured instantaneous frequency $f(t)$ (black dots) and voltage spikes (following same color code). The measured self-consistent values f^* during the working memory are in agreement with the respective geometrical solutions.

Similarly as shown for the mathematical models, the hardware one also has a relatively large region of parameter space where there is bistability, as shown in the Supplementary Fig. 11

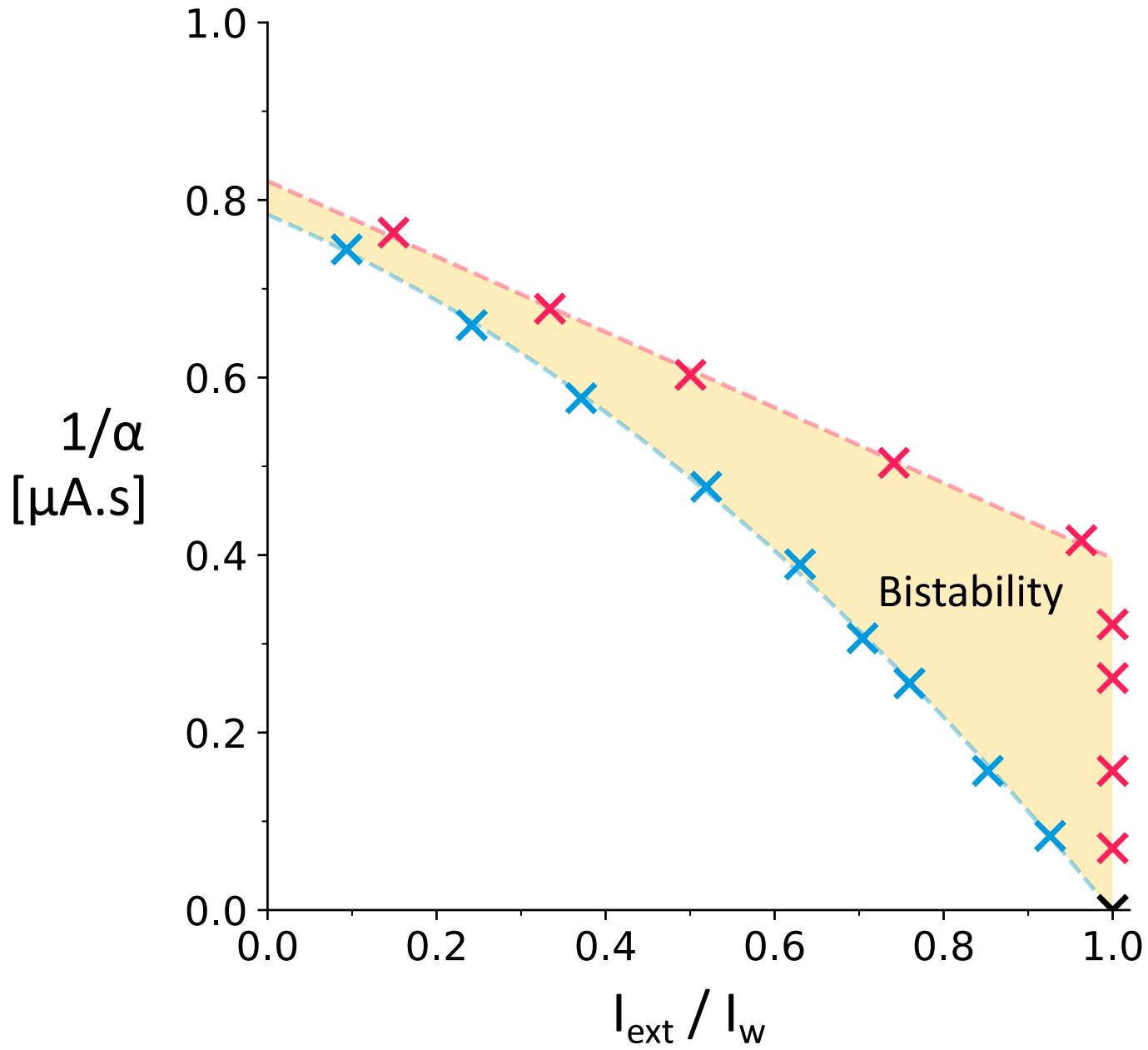

Supplementary Figure 11: Hardware Model Bistability Phase Diagram. The region of bistability is shown in yellow in the $I_{ext} - (1/\alpha)$ plane, where the current I_{ext} is normalized to the threshold current $I_{min} \approx 42\mu$A. This threshold is the minimal amount of current for the electronic neuron to produce spikes. α is the same as in (16) and computed from the data ($1/\alpha = \overline{I_w}/f$). In practice, the feedback strength ($1/\alpha$) is controlled through the resistor R_α (cf Supplementary Fig. 9). Crosses indicate measured data points, and the dashed lines are included as a guide to the eye.

G. Computational Efficiency and Scaling

The scaling is as follows. Consider two populations, E and I, containing N_E and N_I neurons, respectively. Each population is fully recurrently connected, resulting in approximately $N_{s,E} \sim N_E^2$ and $N_{s,I} \sim N_I^2$ synapses. In addition, the two populations are interconnected in an all-to-all manner, yielding $N_{s,EI} \sim N_E N_I$ synapses in each direction. For simplicity and definiteness, we adopt the customary 4:1 ratio between excitatory and inhibitory neurons (80% and 20%, respectively). Denoting the total number of neurons by $N = N_E + N_I$, the total number of synapses (intra- and inter-population combined) is:

$$N_s \sim (0.8^2 + 0.2^2)N^2 + 2(0.8 \times 0.2)N^2 = N^2. \tag{43}$$

Therefore, the network contains approximately N neurons and N^2 synapses.

Assuming a leaky integrate-and-fire (LIF) neuron model and an exponential synaptic-current model, the number of numerical operations required to update each neuron or synapse is comparable (~ 5 floating-point operations (flops) according to Ref. [26]), since both are described by first-order linear differential equations. We denote this computational cost by $N_{\rm op}$. The total number of operations required for the numerical simulation of two E–I populations implementing a working-memory function is therefore of the order of:

$$N_{\rm op}(N^2 + N) \sim N_{\rm op}N^2, \tag{44}$$

for large N. In contrast, the number of operations required by our minimal model, consisting of a single neuron with a self-excitatory synapse, is approximately $2N_{\rm op}$. Consequently, even for relatively small populations of the order of 10 neurons, the computational efficiency gain is already approximately two orders of magnitude.

In theoretical studies, simulations of neuronal populations may be necessary to investigate specific features of the emergent dynamics of complex networks. In hardware implementations, however, the primary objective may simply be the realization of a practical cognitive function.

A similar scaling argument applies to neuromorphic hardware. In this context, the relevant cost is not the number of numerical operations but rather the energy dissipated per spike. The estimate follows from the fact that a spike

corresponds to the discharge of a capacitor through a resistor (C_m and R_a, respectively, in our model). The dissipated energy is proportional to:

$$E \sim CV^2. \tag{45}$$

Similarly, in the synaptic circuit, the current impulse $\delta(t)$ generated by each spike is filtered by the $R_w C_w$ network and subsequently dissipated. Thus, the total energy dissipated per spike is approximately:

$$E \sim (C_m + C_w)V^2. \tag{46}$$

In mixed-signal very-large-scale integration (VLSI) neuromorphic processors, such as Dynap-SE, the use of neuronal populations is often necessary to mitigate device-to-device variability arising from transistor mismatch. Interestingly, Dynap-SE implements adaptive exponential integrate-and-fire (AdEx) neurons, which include a slow self-inhibitory adaptation current.
