# Supplementary material for: A minimal model of working memory in neural systems and neuromorphic circuits
Source: Nat Commun. 2026 Aug 17;17:9867. doi: 10.1038/s41467-026-75704-3 (PMC13578268; doi:10.1038/s41467-026-75704-3)
Supplement: Supplementary file 2 [file 41467_2026_75704_MOESM2_ESM.pdf]

nature portfolio

Peer Review File

A minimal model of working memory in neural systems and neuromorphic circuits

Corresponding Author: Dr Damien Depannemaecker

This file contains all reviewer reports in order by version, followed by all author rebuttals in order by version.

Attachments originally included by the reviewers as part of their assessment can be found at the end of this file.

Version 0:

Reviewer comments:

Reviewer #1

(Remarks to the Author)
Please see attached file.

(Remarks on code availability)
There was no code provided.

Reviewer #2

(Remarks to the Author)
General comment:
To enable large-scale numerical simulations, the authors propose a minimal modification (single sign-change) to phenomenological neuron models (Izhikevich, AdEx, aQIF) that enables persistent activity for working memory. The authors also demonstrate the generality of the principle spanning from single neurons to networks, and provide hardware implementation. However, the work requires substantial additional effort to make it rigorous. The software simulations, hardware implementation, and analysis remain superficial and lack the depth needed to support the strong claims made in the Introduction and Conclusion. I have the following suggestions.

1. Motivation and related work
a. The Introduction cites several previous theoretical single-neuron models that implement a stable persistent state only from 1996-2002 [7, 12, 14, 21]. The authors also claim their model is the first numerically efficient phenomenological model for working memory, but discussion of work from past twenty years is missing.
b. At line 62-63, the authors argue that "there is no phenomenological single neuron model that describes in a numerically efficient manner a working memory function". However, they do not mention what current neuromorphic systems/chips and large-scale simulations use to implement working memory. Do they use populations of neurons, other numerically inefficient neuron models, or do they not bother implementing working memory at all? In a word, what is the current practice?
c. The Izhikevich, aQIF, and AdEx models have been used for tens of years. The "single sign-change" the authors made sounds like an obvious modification. Could the authors explain what prevented people from thinking of this?
A more up-to-date and in-depth related work review would highlight the novelty and significance of the work.

2. Quantitative evaluation
The authors argue that their minimal model is the first phenomenological single-neuron model describing working memory in a numerically efficient manner. However, this claim is supported only by several high-level sentences in the Introduction without quantitative evaluation. Comparing computational cost and other aspects against existing neuron and network models is important for readers to assess how superior the proposed minimal model actually is and understand why the model enables large-scale simulations.
Additionally, the evaluation would be more complete with hardware implementation comparison.

3. Large-scale numerical simulations/applications

The authors claim that their model enables large-scale modeling of, for example, epilepsy and cortex, as well as applications in brain-machine interfaces, robotics, and edge AI devices. However, the current simulations are proof-of-concept demonstrations and the largest simulation in the manuscript is a 5,000-neuron synthetic network. Can previous models of working memory achieve similar scale by adopting approaches like mean-field? The work would be more practical by either providing comparisons showing the proposed model enables large-scale simulations that were previously infeasible or demonstrating its application to a real-world task.

Other minor comments:
1. Readability
a. Line 62-63, the sentence "However, to the best of our knowledge, there is no phenomenological single-neuron model that describes in a numerically efficient manner a working memory function" is an important argument for motivation but its word order is awkward.
b. Line 66, " corresponds to transform" should be " corresponds to transforming".
c. Line 329, "w" should be "Iw".
d. Fig. 7 caption, "reported in I" should be "reported in Table I".

2. The authors present three extension neuron models (eQIF, eAdEx, eIzhikevich) with equations and response figures showing that each model works for modeling working memory. However, the authors do not compare these three variants. An analysis would help readers understand the trade-offs and guide model selection for different tasks.

3. The caption of Fig. 1 lists $\Delta T = 2mV$, but this parameter does not appear in Eqs. (1) to (4).

4. Lines 145-146 refer to "the left panel of Figs. 1 and 2" demonstrating "stable working memory cognitive function in the eQIF model." Do authors mean "left half/portion"? "left panel" is unclear because the figures have vertical layouts. Additionally, Fig. 1 does not use eQIF model and does not demonstrate stability.

5. Fig. 1 lists all parameters used in its caption, but Fig. 2 does not. Do they share the same set of parameters?

6. Please add clearer legends to improve figure visibility if applicable, particularly for Figs. 3-6.

7.Please emphasize what new findings Section II.B provides compared to Section II.A, as both analyze bistability. Additionally, Figs. 5 and 6 could be integrated into a single multi-panel figure.

8. The actual hardware implementation is an important contribution.
a. I suggest authors move the circuit schematic (Fig. 11) to the main text, rather than Appendix.
b. The authors do not discuss how the circuit design changes correspond to the "single sign-change" in the theoretical model. If the circuit modifications are also moderate, is it feasible to implement a configurable neuron circuit that can switch between working memory mode and adaptation mode? This would be a more generalizable hardware contribution.

9. The Conclusion would benefit from adding discussion of limitations and future work.

(Remarks on code availability)
There is no code shared.

Version 1:

Reviewer comments:

Reviewer #1

(Remarks to the Author)
The authors have addressed most of my points. The presentation and organization of the article is much better than the previous version. However, the details added about the circuit have raised some further issues that need to be addressed. There are also some other minor issues.

Major Question
Sections A, B and D (and the appendix) focus on a model (1) where F(V) is a nonlinear function of V. The nonlinearity in F(V) is important as it means the model has two equilibrium points, one of which is unstable. This is what enables the bistability between the equilibrium point and the spiking solution which is crucial for the working memory model.

There is a disconnect between this and the model for the circuit. In section C, no model equation is given for dV/dt. However, the paper says that "the behaviour is analogue to the simple LIF". There must be something more to the system. A simple LIF model, even with the addition of the extra variable Iw, could not produce the type of hysteresis observed. The LIF model only has one equilibrium point which is either stable or unstable depending on the value of Iext. The model for V needs to be included with some explanation. Presumably this is available in one of the cited papers.

A more minor point is that Eq. (11) is not the same as Eq. (6), as H(V) is used differently in the two equations. In Eq. (6) H(V) is a linear function of V which is part of the differential equation, while Eq. (9) defines the reset to Iw when there is a spike. In

Eq. (11), H(V) (as defined by eq. (12)) is a representation of reset to Iw. So for the model of the circuit, H(V)=0 to follow the notation of Eq. (6) I suggest H(V) should be called something else in Eq. (11), or just put Eq. (12) directly in Eq. (11). I further suggest that the Appendix include an analaysis of the QIF model with Eq. (6) having H(V)=0. This would show that there is no need for direct dependence on V in the equation for IW, given a better link between the QIF model and the circuit.

Minor Points
Fig 1-2 State all parameter values for model.
Fig 3. Indicate the values of a and b in each subplot
Fig 5. Indicate the value of Iext in figure caption and/or main text when describing figure.

(Remarks on code availability)

Reviewer #2

(Remarks to the Author)
We thank the authors for their revisions. The manuscript has improved in presentation and coverage of related work and background.

One suggestion on the response letter: it would help to briefly summarize the specific changes made to the manuscript at the end of each response. This would make the revision much easier to follow.

We have the remaining concerns:

1. The central claim of this paper is that it provides the first numerically efficient phenomenological single-neuron model of working memory. When we raised this in the previous round, the authors pointed to the Izhikevich 2004 paper without providing direct numbers. We think this is worth doing properly. Before this work, working memory required two populations of neurons (one excitatory, one inhibitory). A direct report of the computational cost of this conventional approach versus the proposed single-neuron model would make the efficiency argument concrete and convincing. The authors can either extract the relevant numbers from the cited reference or compute them directly.
Additionally, the authors have already built a hardware implementation, which puts them in a good position to make the same comparison at the circuit level. This hardware comparison, combined with the software one, would give readers a clear sense of what "minimal" actually means in practice, and would substantially strengthen the paper's central contribution.

2. The authors demonstrate distractor robustness using a fixed perturbation amplitude, but this is not enough to support a general robustness claim. For exmaple, how large a distractor can the system tolerate before the memory is lost and how does noise affect the persistent state? A more systematic analysis of these questions would be needed to establish the robustness of the proposed working memory mechanism.

(Remarks on code availability)
We thank the authors for making the repository public. However, it lacks a README file, documentation, and usage demonstration.

Version 2:

Reviewer comments:

Reviewer #1

(Remarks to the Author)
The authors have adequately addressed my concerns. There are just a few minor issues, listed below. After these are addressed the manuscript will be ready for publication. I do suggest the authors make a thorough proof reading of the manuscript to look for other typos.

- Is there a relationship between R_m in eq. (10) and g_m in eq. (11)? If so please indicate this. If they are independent, please give the value of g_m in the caption for Fig. 5

- There is a blank line labelled as eq. (12). The label needs to be removed.

- A few lines below eq. (13) "excitability do a" -> "excitability due to a"

- H(V) in eq. (14) should be B(V)

(Remarks on code availability)

Reviewer #2

(Remarks to the Author)
We thank the authors for their revisions and are satisfied with the responses provided. However, we hope the authors will carefully check the revised manuscript during proofing to ensure that all promised changes have been properly included.

In particular, the computational efficiency comparison (the N^2 scaling argument and hardware energy comparison) promised in the response cannot be found anywhere in the revised manuscript, and no GitHub repository URL is provided in either the manuscript or the response letter.

(Remarks on code availability)
No GitHub repository URL is provided.

Open Access This Peer Review File is licensed under a Creative Commons Attribution 4.0 International License, which permits use, sharing, adaptation, distribution and reproduction in any medium or format, as long as you give appropriate credit to the original author(s) and the source, provide a link to the Creative Commons license, and indicate if changes were made.
In cases where reviewers are anonymous, credit should be given to 'Anonymous Referee' and the source.
The images or other third party material in this Peer Review File are included in the article's Creative Commons license, unless indicated otherwise in a credit line to the material. If material is not included in the article's Creative Commons license and your intended use is not permitted by statutory regulation or exceeds the permitted use, you will need to obtain permission directly from the copyright holder.
To view a copy of this license, visit https://creativecommons.org/licenses/by/4.0/

1- Reply to Reviewer #1

We thank Reviewer #1 for the positive evaluation of our work, which is assessed as "interesting". The reviewer also found the organization and presentation of insufficient quality for Nat Comm. We accept this criticism and we would like to thank the Reviewer for valuable and constructive comments and for the suggestions made, which allowed us to improve our manuscript. We provide below detailed replies to all the points raised (Reviewer comments in blue, replies in black).
We hope that the referee will be satisfied by our answers and the changes made in the presentation and will recommend the publication of our work.

Reviewer #1
Section IIA presents a rather poorly justified back-of-the envelope calculation as to why models with the excitatory current have this bistability property. In particular, more justification and statement of underlying assumptions for eq.(10) are needed. (E.g., as shown later on the spiking solution, Iw periodically varies between two values, so what does Iw in eq. (10) represent? Minimum, maximum, mean?). Also how was the stability of the intersection points in Fig. 3 shown?
Section IIB presents an analysis of one of the example models, which shows in a more methodical way why the bistability occurs, although some of the steps and methodology are not clear. In a sense this section makes the previous one redundant. All that is needed is to show bistability in the model and this can be done via phase plane and bifurcation analysis. A clear presentation of how this occurs for the eQIF model in the main body of the text and diagrams such as Fig. 3 and Fig. 5 for the other two models in the appendix, could then be followed up with a discussion of the situation for a general model.

We thank the Reviewer for these valuable comments. We have followed the advice of the Reviewer and reorganized these two sections of the paper. We now firstly illustrate in one figure the working memory and bistability (hysteresis) in the minimal eQIF model (eQIF). Secondly, we provide an improved formal analysis of the model and a better description of the phase plane structure, showing the bistability. On the other hand, we moved and improved discussion of the geometric construction to the experimental section with the circuit data. This emphasizes the generality of our finding. This last point is further justified by the simulation results of the AdEx and Izhikevich models, modified from self-inhibition to self-excitation, that we show in the Appendix.

Reviewer #1
The ideas could be strengthened if some discussion of how large (with respect to Iext) the region of bistability the model needs to be in order for working memory to be viable. In Fig. 6 the region seems quite large. If parameters are varied so that this is smaller is the working memory still robust?

This is an important point, we thank the reviewer for pointing it out. Indeed the region of bistability is very large and it does not require fine tuning as is the case in the conventional AdEx and Izhikevich models. To address this point, we now explicitly compute the boundaries of the bistable region for different parameter combinations of the model (see

Figs.2c and Fig.9b). This analysis demonstrates that the bistable regime occupies a substantial portion of the parameter space and does not rely on fine tuning.
In particular, the persistence of bistability across distinct parameter configurations highlights the robustness of this dynamical feature.
We also show this for the circuit model, which provides an additional strong justification for the lack of fine tuning the working memory requires. We are now showing a few cases for different parameters in the Supplemental Material.

Reviewer #1
Section IIC presents a network version of the model and corresponding mean-field model. The key point seems to be that the network inherits the properties of the single neuron, i.e., it can store a single memory. I don't see why this is interesting. If the network were able to store many memories that might be interesting. In fact, contrary to the statement on p. 14 the reason many people study mean-field models is not because they replicate the behaviour of the single neuron but to show how networks can exhibit new behaviour not possible in the single neuron model.

We accept this criticism and that our message was not clear. We have now improved the discussion. Our point was to demonstrate that a purely excitatory network made of self-excitatory neurons: (i) remains stable and (ii) keeps the work memory function. Then, we showed that, similarly to Montbrio et al, we may obtain a mean field model that faithfully represents the simulated network behavior.
With regard to the question of emergent network behavior in contrast to the single neuron, that is not the focus of our work. Indeed exploring the possibility of obtaining persistence of subnetworks would be an interesting direction to pursue. Nevertheless, there is some genuine new behavior that is expressed in the network (and captured by the MFM), which is not possible for a single neuron. This concerns the observation of synchrony. This is prominent in the initial moments, right after the network is excited, but it rapidly averages out. We have now discussed this feature in more detail. By means of sorting, we made the interesting observation that synchrony persists within partitions of the network, i.e. within subnetworks. The presence of synchrony and its decay in a fully excitatory network is of interest in research of ictal dynamics in epilepsy.

Reviewer #1
Section IID presents experiments of a circuit implementation of. This is very interesting, but they should have mentioned that hardware schematic is in Appendix C.

We are very glad that the Reviewer appreciates this original aspect of our work. Indeed, we are trying to pursue an original direction by developing and implementing a new type of hardware, which, in contrast to all other approaches, is fully analog, minimal (i.e. simple) and bio-inspired. We hope it will eventually open the way for a novel approach to neurocomputing. We apologize for overlooking mentioning the schematic of the circuit. Following the suggestion of Reviewer #2 we have now moved it to the main text.

• p.8 line 145 refs to left panel of Figs 1 and 2 but they have no left panel.

We thank the Reviewer for pointing out this type, due to an earlier version of the manuscript.

• p.6, line 130. IKCa is not excitatory current. Since it is a potassium current it slows down or prevents spiking.

The Reviewer is right. We apologize for this mistake. We are now mentioning the L-type Ca-current in smooth muscle cells, as an additional example besides the I_T.

• p.10 How is red curve in Fig 3 computed? The frequency formula is exact for steady state firing frequency of QIF model, so I don't understand why you say the formula provides a "good fit" of the FI curve. Might be better stated the numerical method that is providing a good replication of the exact formula.

The Reviewer is correct. The response of a leaky-integrate and fire type of model is exact and it is the numerical model that approximates well the behavior. This mistake is fixed, we now use the formula to fit the experimental measurements of our memristive neuron circuit behavior. Thank you for this comment.

• p.11 It is not clear why the paper brings up the fact that the model changes from type I to type II depending on whether the applied current is increasing or decreasing. Is/how is this relevant?

It was just an observation that the nature of the critical points, how one emerges and how the other disappears is different, which is another feature of the hysteresis. We, nevertheless, followed the advice of the Reviewer and erased it for the sake of clarity in the presentation.

• p.11 Guttman, Lewis & Rinzel (1980) J. Physiol. 305: 377-395 show a figure of an experiment on the squid giant axon that is very similar to Fig. 4(a). It is worth citing as an example of a neuron that exhibits bistability.

Thank you very much for this great reference, which we now mention in the manuscript.

In addition, the referee may find it interesting that our electronic neuron model also beautifully captures the second figure in p.11 of Guttman et al (1980), where the input is increased and the spiking becomes interrupted, while the hysteresis effect persists. This is qualitatively identical to the results that we obtained and showed in the photo of the oscilloscope below. The green trace is the triangular input, and in yellow the spiking activity of the neuron with self-excitation. Note that the hysteresis is also clearly observed in the asymmetry of the onset of spiking and the end. The reason for the interruption of spiking at the upper part of the triangle is that the input current to the neuron becomes so large that the neuron circuit enters the equivalent of the "depolarization block". In fact, one may see in the response of the electronic neuron (right panel from Wu et al Phys Rev App 23, 034030 2025), if the current is too strong the spiking stops. The physical reason is in the fact that when the thyristor remains conducting if the input is above the "holding" current, it then represents a large leak and there are no more spikes.

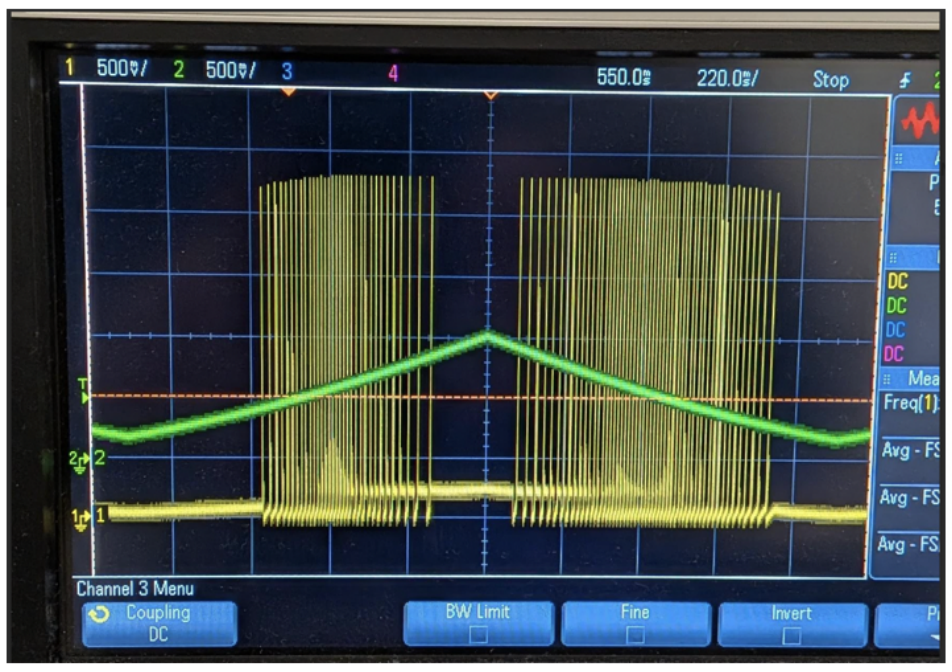

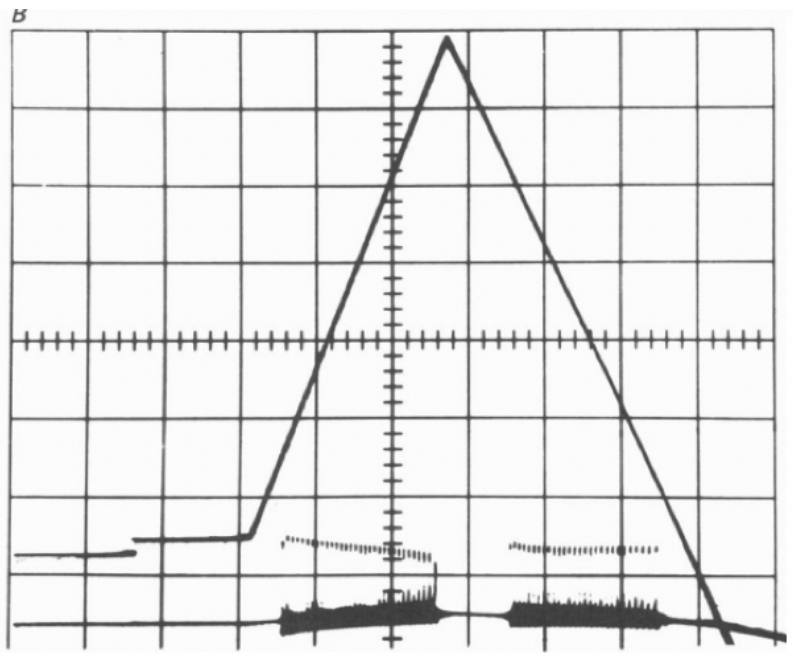

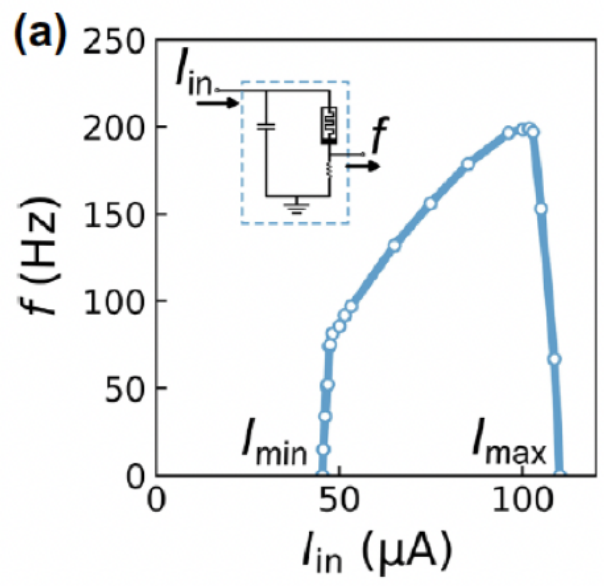

• p.13 What are the numerical packages/methods used to generate Figs. 5 and 6? I assume that the variability in the height of the APs in Fig. 6 is due to numerical method? How were the limit cycles in Fig. 6 generated?

The numerical simulations were done using the Brian2 package. This is now mentioned in the text. Thank you for this comment.

• p.13 Fig. 5 would be improved by showing trajectories for two initial conditions illustrating the bistability.

Thank you for the suggestion. The original Fig. 5 only considered the case Iext=0Iext=0, which does not exhibit bistability. The figure now consists of three panels corresponding to different parameterizations of the self-excitatory equation. In particular, we include a case in which bistability emerges and explicitly illustrate it by plotting two distinct trajectories converging to different stable states in the phase plane, one converging to the resting (relaxation) state and the other evolving toward sustained spiking activity.

• p.13 “Obtain persistence in large ...” persistence of what?

Thank you for pointing out this omission. It should read persistence of a spiking state

• p.14 Don’t need intrinsically bursting neurons to get population bursts. Quite the opposite. The key point of several studies is that population bursting arises when intrinsically spiking neurons are coupled together, i.e., it is a network induced phenomenon.

The Reviewer is correct, this part is now rewritten.

• p.16 eq. (13) gsyn,i should be gsyn

Thank you. This is now fixed.

Once again, we thank the Reviewer #1 for the very constructive comments and the careful reading of our manuscript. We are confident that following their advice the presentation has been substantially improved and that the Reviewer will recommend the publication of our work.

Reply to Reviewer #2

We thank Reviewer #2 for the overall positive evaluation of our work. Below we provide detailed answers to all the points made. (Reviewer comments in blue, replies in black).

Reviewer #2 (Remarks to the Author):
General comment:
To enable large-scale numerical simulations, the authors propose a minimal modification (single sign-change) to phenomenological neuron models (Izhikevich, AdEx, aQIF) that enables persistent activity for working memory. The authors also demonstrate the generality of the principle spanning from single neurons to networks, and provide hardware implementation. However, the work requires substantial additional effort to make it rigorous. The software simulations, hardware implementation, and analysis remain superficial and lack the depth needed to support the strong claims made in the Introduction and Conclusion.

We thank the Reviewer for the careful reading and the many constructive comments. We have carefully taken them into account and followed their advice to improve the presentation and deepen our arguments and analysis.

Reviewer #2
I have the following suggestions.
1. Motivation and related work
a. The Introduction cites several previous theoretical single-neuron models that implement a stable persistent state only from 1996-2002 [7, 12, 14, 21]. The authors also claim their model is the first numerically efficient phenomenological model for working memory, but discussion of work from past twenty years is missing.

As far as we can tell, in the past twenty years the work on working memory has only considered the case of populations. Notably, the numerical work of Compte et al. [8] and as discussed in the popular 2014 textbook by Gerstner et al. [11]. The more recent work has been described in the review of Barak & Tsodyks (2014) and in Constantinidis et al (2018), among others. In recent years new mechanisms of persistent activity have been proposed, such as based on short term synaptic plasticity, and there remains a certain debate which is outside the scope of our work.

Durstewitz, D., Seamans, J. & Sejnowski, T. Neurocomputational models of working memory. *Nat Neurosci* 3 (Suppl 11), 1184–1191 (2000). https://doi.org/10.1038/81460
Science. 2008 Mar 14;319(5869):1543-6. doi: 10.1126/science.1150769.
Synaptic theory of working memory
Gianluigi Mongillo, Omri Barak, Misha Tsodyks
REVIEW Chaudhuri R, Fiete I (2016) Computational principles of memory. Nat Neurosci 19:394–403. 10.1038/nn.4237
REVIEW Zylberberg J, Strowbridge BW (2017) Mechanisms of persistent activity in cortical circuits: possible neural substrates for working memory. Annu Rev Neurosci 40:603–627. 10.1146/annurev-neuro-070815-014006
debate:

for persistent firing J Neurosci. 2018 Aug 8;38(32):7020–7028. doi: 10.1523/JNEUROSCI.2486-17.2018 this is a **good review like**
Persistent Spiking Activity Underlies Working Memory
Christos Constantinidis

We also provide, as a general reference of recent work, the very recent 2025 textbook by XJ Wang, who is an expert in the field. While the book discusses working memory in detail, we failed to find there references to single-neuron numerically-efficient phenomenological models. So we conclude that the recent literature is surprisingly scarce along this line.

Reviewer #2
b. At line 62-63, the authors argue that "there is no phenomenological single neuron model that describes in a numerically efficient manner a working memory function". However, they do not mention what current neuromorphic systems/chips and large-scale simulations use to implement working memory. Do they use populations of neurons, other numerically inefficient neuron models, or do they not bother implementing working memory at all? In a word, what is the current practice?

The reviewer has the right intuition, the current research considers networks and it is conventional wisdom (cf textbook Neuronal Dynamics, by Gerstner et al) that two populations are required, one excitatory and one inhibitory. This is because conventional wisdom also dictates that the activity of a purely excitatory network will run away, hence an inhibitory population is needed as balance (cf previous reference). All the individual neurons used in those network models are simple spiking neurons, leaky-IF, quadratic-IF or exponential-IF (cf previous reference and, for instance, Compte et al). What we showed is that, contrary to this conventional wisdom, such single neuron models with a *self-excitatory* intrinsic current can exhibit the working memory cognitive function. Moreover, we also showed that networks of those single neurons are also *stable* and can show working memory, although no inhibition (or self-inhibition) is present.

Regarding the question of "neuromorphic systems/chips", we are glad the referee asked this question. In fact, we think that our work may influence future designs. Specifically, the DYNAP-SE2 CMOS VLSI chip is based on neurons that implement the AdEX model, i.e. an exponential-IF plus an self-inhibitory intrinsic current. We demonstrate that in the AdEx model a single change of sign (i.e. a single change in the current direction in the chip) allows them to implement a working memory (i.e. persistent spiking). Hence, a small change in the design would enlarge the potential behavior and applicability of the chip cognitive functions and behaviors.

We have added a paragraph to address this point.

Reviewer #2
c. The Izhikevich, aQIF, and AdEx models have been used for tens of years. The "single sign-change" the authors made sounds like an obvious modification. Could the authors explain what prevented people from thinking of this?

We fully agree with the Reviewer, this seems an obvious modification and is an intriguing question. A plausible answer can be found from reading the Introduction of the excellent book by Izhikevich "Dynamical Systems in Neuroscience". There, the goal was to deduce the simplest model realizing all neuron *firing patterns*. He used as reference of the biorealistic Hodking-Huxley and other models that also include adaptation current. This led to the simple model defined in Eqs. 8.3 and 8.4. By changing the sign in Eq. 8.3 (-u -> +u) one does *not find a new firing pattern*, it is still tonic spiking. However, what one now finds by the sign change is a *large* regime of bistability (quiescent and tonic spiking) where a robust working memory *function* can be realized. Hence, it is not a new firing pattern but a *new behavior, or cognitive function*. We may also mention that bistability can be found in the original Izhikevich model, but only in a *tiny region*, hence requires a fair amount of *fine tuning*, which renders it impractical.

A more up-to-date and in-depth related work review would highlight the novelty and significance of the work.

We have added an improved discussion of network models.

Reviewer #2
2. Quantitative evaluation
The authors argue that their minimal model is the first phenomenological single-neuron model describing working memory in a numerically efficient manner. However, this claim is supported only by several high-level sentences in the Introduction without quantitative evaluation. Comparing computational cost and other aspects against existing neuron and network models is important for readers to assess how superior the proposed minimal model actually is and understand why the model enables large-scale simulations.

The computational cost of our model (measured in floating point operations) is identical to that of Izhikevich, since it only concerns a change of sign. The comparison of computational cost of phenomenological versus biorealistic models has been discussed in the paper by Izhikevich (2004) "Which model to use for cortical spiking neurons?" that counts more than 3500 citations. We have now mentioned this in the manuscript.

Additionally, the evaluation would be more complete with hardware implementation comparison

The only reasonable comparison would be with DYNAP-SE2, which is also an analogue hardware implementation of a spiking neuron. However, to our knowledge, a working memory function has not been implemented with that chip.

Reviewer #2
3. Large-scale numerical simulations/applications
The authors claim that their model enables large-scale modeling of, for example, epilepsy and cortex, as well as applications in brain-machine interfaces, robotics, and edge AI devices. However, the current simulations are proof-of-concept demonstrations and the largest simulation in the manuscript is a 5,000-neuron synthetic network. Can previous

models of working memory achieve similar scale by adopting approaches like mean-field? The work would be more practical by either providing comparisons showing the proposed model enables large-scale simulations that were previously infeasible or demonstrating its application to a real-world task.

The claim that our model enables large-scale modeling is supported by the argument presented above in (2). Namely, that our type of model allows for efficient numerical simulations due to their small computational cost while keeping non-trivial dynamics. In this regard, our contribution is that our model adds a relevant cognitive function (persistent spiking, working memory, dynamical attractor) to the repertoire of the numerically efficient models. In regard to potential applications, we may mention that self-excitation and mutual excitation are expected to be relevant for studies in epilepsy. In fact, a line of research is concerned with the implementation of "virtual twin brain" models, which are expected to provide guidance in neurosurgical ablations. These are so called "whole brain models" and naturally require large scale numerical simulations, so phenomenological models are in order. More specifically, there is the "epileptor" model (old ref.28) which aims to describe an epileptogenetic node in the brain. The epileptor is modeled with a few differential equations that include a short-term self-excitation (like ours) and a longer term self-inhibition. Hence, our simulations of networks of purely excitatory synaptic interactions (including self-interaction) that overcome an initial global synchronous state and become partitioned into smaller regions may be of interest and useful for those kinds of studies. We added a paragraph: *Beyond the proof-of-concept simulations presented here, the relevance of the proposed framework for large-scale applications should be considered in relation to canonical models of working memory and seizure dynamics. Large-scale implementations have often relied on mean-field or neural mass reductions to achieve tractable simulations at cortical or whole-brain scales [34, 50]. Similarly, seizure modeling has benefited from dynamical systems approaches, such as neural field or neural mass frameworks, where pathological transitions are interpreted as bifurcations between distinct dynamical regimes [52, 53]. From a dynamical systems perspective, both working memory maintenance and epileptic seizures can be viewed as transitions between metastable or attractor states within recurrent circuits; however, they differ in their dynamical signatures. Working memory typically corresponds to stable persistent firing states characterized by moderate, sustained activity, whereas seizures involve transitions toward regimes of abnormally elevated firing rates, increased neuronal synchrony,and reduced dynamical variability, often associated with large-scale recruitment of neuronal populations. Existing large-scale models frequently achieve scalability by sacrificing detailed spiking dynamics or simplifying intrinsic mechanisms underlying persistence and excitability. In contrast, the present minimal formulation preserves an explicit spiking description while remaining compatible with analytical mean-field reductions, thereby offering a bridge between microscopic neuron dynamics and macroscopic population models.*

Reviewer #2

Other minor comments:
1. Readability
a. Line 62-63, the sentence "However, to the best of our knowledge, there is no phenomenological single-neuron model that describes in a numerically efficient manner a working memory function" is an important argument for motivation but its word order is awkward.
b. Line 66, " corresponds to transform" should be " corresponds to transforming".
c. Line 329, "w" should be "Iw".
d. Fig. 7 caption, "reported in I" should be "reported in Table I".

We thank the reviewer for pointing out these issues and typos. They have been corrected.

2. The authors present three extension neuron models (eQIF, eAdEx, e Izhikevich) with equations and response figures showing that each model works for modeling working memory. However, the authors do not compare these three variants. An analysis would help readers understand the trade-offs and guide model selection for different tasks.

The three models are similar and they are all widely used in computational neuroscience. The QIF and Izhikevich have a non-linear term in the membrane potential equation that is a quadratic polynomial. They merely differ in the choice of its parameters. The QIF is the simplest choice, best for mathematical studies. The Izhikevich is more flexible and can capture more biological behaviors by choosing the coefficients. The AdEx also captures multiple behaviors, but its non-linearity is exponential which is argued to better mimic biological neuron traces. We have included a sentence to more explicitly refer the reader to the relevant textbooks and literature, which were already cited [old ref.11 and 41].

3. The caption of Fig. 1 lists ΔT = 2mV, but this parameter does not appear in Eqs. (1) to (4).

This is now fixed.

4. Lines 145-146 refer to "the left panel of Figs. 1 and 2" demonstrating "stable working memory cognitive function in the eQIF model." Do authors mean "left half/portion"? "left panel" is unclear because the figures have vertical layouts. Additionally, Fig. 1 does not use eQIF model and does not demonstrate stability.
5. Fig. 1 lists all parameters used in its caption, but Fig. 2 does not. Do they share the same set of parameters?
6. Please add clearer legends to improve figure visibility if applicable, particularly for Figs. 3-6.
7.Please emphasize what new findings Section II.B provides compared to Section II.A, as both analyze bistability. Additionally, Figs. 5 and 6 could be integrated into a single multi-panel figure.

We apologize for these various mistakes and shortcomings, we have now thoroughly improve the figures and their captions.

8. The actual hardware implementation is an important contribution.
a. I suggest authors move the circuit schematic (Fig. 11) to the main text, rather than

Appendix.
b. The authors do not discuss how the circuit design changes correspond to the "single sign-change" in the theoretical model. If the circuit modifications are also moderate, is it feasible to implement a configurable neuron circuit that can switch between working memory mode and adaptation mode? This would be a more generalizable hardware contribution.

We thank the Reviewer for the positive appreciation of our hardware implementation. Following the suggestion we have improved the clarity of the circuit diagram to be accessible to non-experts and moved it into the main text. The Reviewer has the right intuition, indeed the circuit can be selected to do self-excitation (ie working memory) or adaptation by a simple choice of the output pin, which selects the polarity of the current (positive for excitation and negative for inhibition).

9. The Conclusion would benefit from adding discussion of limitations and future work.

We followed this advice and improved the Conclusion in those two aspects.

We would like to thank again the Reviewer #2 for the many useful and constructive comments. We believe that this advice has helped us to significantly improve the manuscript and hope that the Reviewer will recommend its publication

Reviewer #2 (Remarks on code availability):

There is no code shared.
The code is now available on GitHub:
https://github.com/GabrieleCasagrande/MinimalNeuro_WorkingMemo

Dear Editor,

We are pleased to resubmit our manuscript entitled *“A minimal model of working memory in neural systems and neuromorphic circuits”* and would like to thank you and the reviewers for the time and care devoted to its evaluation. We have carefully addressed all the comments raised during the review process and believe that the manuscript has been significantly improved as a result.

We have clarified the description of the circuit model by explicitly including the governing equation for the membrane potential and providing additional explanations linking the hardware implementation to conductance-based models. We also revised the notation to avoid ambiguities and expanded the Appendix with additional analyses, as suggested. Furthermore, all minor points regarding figure annotations and parameter reporting have been corrected. We have also extended the analysis of robustness by including a more systematic study of the effects of distractors and noise on the persistence of activity.

We are grateful for the constructive feedback, which has helped us improve the clarity, rigor, and impact of our work. We hope that the revised manuscript now meets the standards for publication and will be suitable for Nature Communication journal.

Thank you for your consideration.

Sincerely,

Damien Depannemaecker (on behalf of all authors)

Reply to the Reviewers

Reviewer #1 (Remarks to the Author):

The authors have addressed most of my points. The presentation and organization of the article is much better than the previous version. However, the details added about the circuit have raised some further issues that need to be addressed. There are also some other minor issues.

We thank the Reviewer very much for the appreciation of our new version and our work in general. We thank again for the previous and the present constructive and useful comments that have helped to significantly improve the clarity of our work. Below we address the new comments in detail.

Major Question
Sections A, B and D (and the appendix) focus on a model (1) where F(V) is a nonlinear function of V. The nonlinearity in F(V) is important as it means the model has two equilibrium points, one of which is unstable. This is what enables the bistability between the equilibrium point and the spiking solution which is crucial for the working memory model.

There is a disconnect between this and the model for the circuit. In section C, no model equation is given for dV/dt. However, the paper says that "the behaviour is analogue to the simple LIF". There must be something more to the system. A simple LIF model, even with the addition of the extra variable Iw, could not produce the type of hysteresis observed. The LIF model only has one equilibrium point which is either stable or unstable depending on the value of Iext. The model for V needs to be included with some explanation. Presumably this is available in one of the cited papers.

We thank the referee for this comment. Indeed the model for V is described in previous work. Nevertheless, following this comment we agree with the Reviewer that a short description would be useful. We have added the equations and the explanation along with an additional panel in the figure. It explains how our hardware model, based on a memristor, is a conductance model in the same spirit as Hodgkin-Huxley and can be most simply rationalized as an analog LIF.

Regarding the Reviewer's claim that a simple LIF plus a slow excitatory variable Iw cannot produce hysteresis, we respectfully disagree. We demonstrate this point by explicit numerical

simulations of the model that we added to the Supplemental Material. We also provide the code in the github. The LIF, having a threshold current to excite spikes, also falls within the assumptions of the geometric solution that we present in Fig.6.
However it is true that the region in parameter space for the bistability is smaller than in models with a non-linearity. But since it is not the main focus of this work, we just added a subsection in the appendix showing the bistability in the LIF model with recurring current.

An interesting further comment in this regard is that the geometric solution also explains why the ReLU model cannot show hysteresis. As its threshold is 0, then one cannot have a subthreshold current. Hence, only the unstable (runaway) solution exists as shown in the figure below.

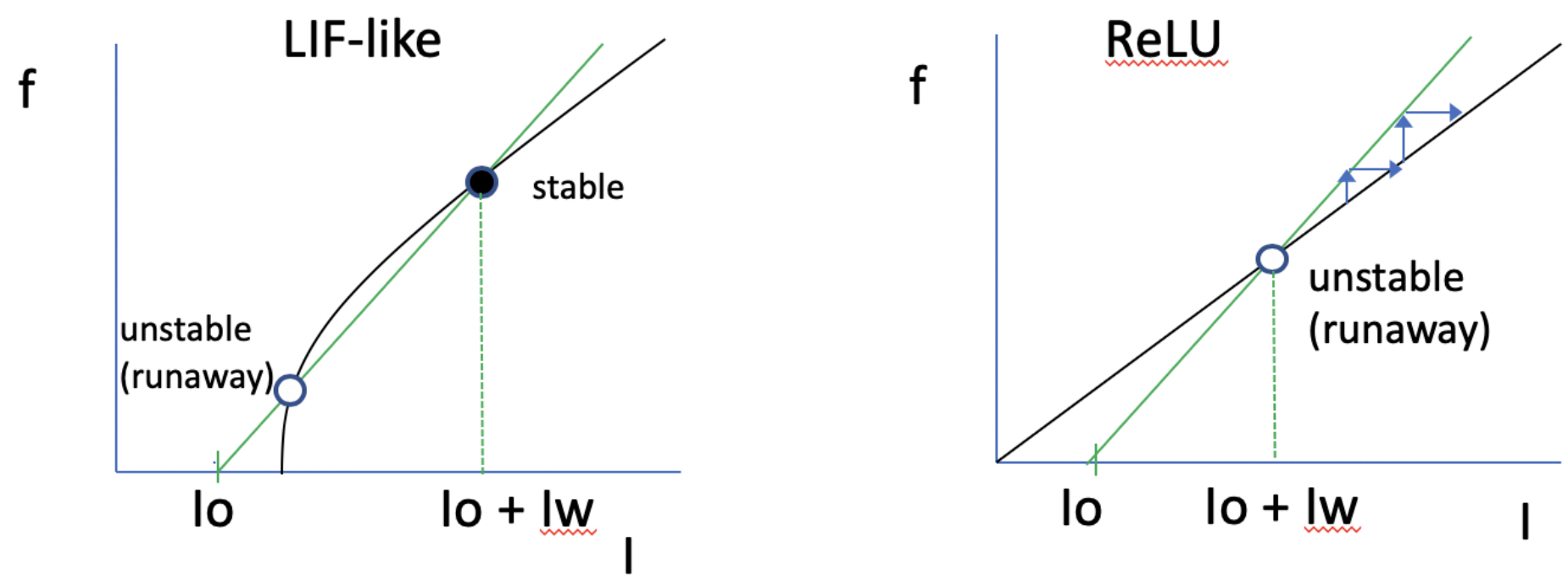

A more minor point is that Eq. (11) is not the same as Eq. (6), as H(V) is used differently in the two equations. In Eq. (6) H(V) is a linear function of V which is part of the differential equation, while Eq. (9) defines the reset to Iw when there is a spike. In Eq. (11), H(V) (as defined by eq. (12)) is a representation of reset to Iw. So for the model of the circuit, H(V)=0 to follow the notation of Eq. (6) I suggest H(V) should be called something else in Eq. (11), or just put Eq. (12) directly in Eq. (11). I further suggest that the Appendix include an analysis of the QIF model with Eq. (6) having H(V)=0. This would show that there is no need for direct dependence on V in the equation for IW, given a better link between the QIF model and the circuit.

We thank the Reviewer very much for this comment. We agree that the choice of H(V) in Eq.11 was not good. We have renamed it as B(V), since it is related to the "current reset" of the mathematical models, namely, Iw <- Iw + b, which is characterized by the "b" parameter.

The Reviewer's suggestion to add the H(V)=0 in the Appendix is also very useful and we have followed the Reviewer's advice. The case H(V)=0 actually serves to clarify the fact that H(V) is not necessary to produce hysteresis. The current reset Iw <- Iw + b is sufficient, as our simulations (and the hardware model) show.

We added a subsection in the appendix to show simulation of the eQIF model under this condition.

Minor Points
Fig 1-2 State all parameter values for model.
Fig 3. Indicate the values of a and b in each subplot
Fig 5. Indicate the value of Iext in figure caption and/or main text when describing figure.

Thank you for all these corrections, that are now taken into account.
Reviewer #2 (Remarks to the Author):

We thank the authors for their revisions. The manuscript has improved in presentation and coverage of related work and background.

It is us that thank you for the constructive and useful comments that helped to improve the presentation of our work.

One suggestion on the response letter: it would help to briefly summarize the specific changes made to the manuscript at the end of each response. This would make the revision much easier to follow.

We apologize for this, but the reason was that we made extensive changes and reorganized the order of the sections. So the highlighted version of the manuscript was almost fully in color, which we considered would not be useful. This time, there are just a few changes, so we are providing the highlighted version.

We have the remaining concerns:

1. The central claim of this paper is that it provides the first numerically efficient phenomenological single-neuron model of working memory. When we raised this in the previous round, the authors pointed to the Izhikevich 2004 paper without providing direct numbers. We think this is worth doing properly. Before this work, working memory required two populations of neurons (one excitatory, one inhibitory). A direct report of the computational cost of this conventional approach versus the proposed single-neuron model would make the efficiency argument concrete and convincing. The authors can either extract the relevant numbers from the cited reference or compute them directly.
Additionally, the authors have already built a hardware implementation, which puts them in a good position to make the same comparison at the circuit level. This hardware comparison,

combined with the software one, would give readers a clear sense of what "minimal" actually means in practice, and would substantially strengthen the paper's central contribution.

We thank the Reviewer for this useful suggestion. We have added a section in the Supplemental Material to describe the estimates.
The scaling is as follows: Let's assume two populations, E and I, with NE and NI neurons each. The populations are fully intra-connected, so each has a number of synapses $NsE \sim NE^2$ and $NsI \sim NI^2$. In addition the populations are interconnected also all-to-all with $NsEI \sim NE*NI$ synapses. For simplicity and definiteness, we adopt the customary 4:1 ratio between NE and NI (80%-20%). Hence if N is the total number of neurons $N=NE+NI$, the total number of (intra plus inter population) synapses $Ns \sim (0.8^2+0.2^2)N^2 + 2*(0.8*0.2)N^2 = N^2$.
Therefore, we have $\sim N$ neurons and $\sim N^2$ synapses.

Assuming a LIF model for the neuron and an exponential model for the synaptic current, the number of numerical operations (~ 5 flops [Izhikevich2004]) is similar for both, as they are first order linear differential equations, which we denote Nop. Hence the total number of operations for a numerical simulation of two E-I populations that implement a working memory, is of the order of $Nop*(N^2+N) \sim Nop*N^2$, for large N. The number of operations that are required for our minimal model, with one neuron and a (synaptic) self-excitation is $\sim 2*Nop$. So even for relatively small populations of the order of 10 neurons, the numerical efficiency is already about two orders of magnitude.

In theoretical work, the simulation of populations may be important to study specific features of the emergent behavior of a complex network. However, in hardware, one may be simply interested in a practical implementation of the "cognitive" function.

The previous scaling applies quite similarly for neuromorphic hardware. However, in this case, the cost is not counted in numerical operations, but rather in energy dissipated per spike. The estimate then follows from the fact that a spike is the discharge of a capacitor onto a resistor (Cm and Ra, respectively in our model). The energy dissipated (equal to the accumulated) is $\sim C*V^2$. Similarly, for the synaptic circuit the current of each spike $\delta(t)$ is transformed by the RwCw filter and then dissipated. Hence for each spike we have a total energy dissipated of $E \sim (Cm + C_w)*V^2$. In fact, in microelectronic (VLSI) neuromorphic chips, such as Dynapse, adopting populations is often a necessity to mitigate the device inhomogeneity problem (mismatch). Interestingly Dynapse only implements AdEx neurons, i.e. with a (self) inhibitory slow current.

2. The authors demonstrate distractor robustness using a fixed perturbation amplitude, but this is not enough to support a general robustness claim. For example, how large a distractor can the system tolerate before the memory is lost and how does noise affect the persistent state? A more systematic analysis of these questions would be needed to establish the robustness of the proposed working memory mechanism.

We thank the Reviewer for the comment. The robustness of the persistent state can be seen from the width of the hysteresis region, which is quite significant and not fine tuned. The time evolution was meant to be mostly a qualitative demonstration. Nevertheless we understand the issue and we have now included in the Supplemental Material a systematic study of the robustness of both the persistent and quiescent behavior against distractor pulses with different strength and duration, which illustrates the point in a more quantitative manner.
Moreover, in the same section in Supplemental material, we also show the effect of the application of additive noise $\delta I(t)$ on top of the distractor pulses.

Reviewer #2 (Remarks on code availability):

We thank the authors for making the repository public. However, it lacks a README file, documentation, and usage demonstration.

Indeed the README;md file was missing. We have now added it to the Github repository with additional information about the provided script and their usage.

REVIEWERS' COMMENTS

Reviewer #1 (Remarks to the Author):

The authors have adequately addressed my concerns. There are just a few minor issues, listed below. After these are addressed the manuscript will be ready for publication. I do suggest the authors make a thorough proof reading of the manuscript to look for other typos.

- Is there a relationship between R_m in eq. (10) and g_m in eq. (11)? If so please indicate this. If they are independent, please give the value of g_m in the caption for Fig. 5

- There is a blank line labelled as eq. (12). The label needs to be removed.

- A few lines below eq. (13) "excitability do a" -> "excitability due to a"

- H(V) in eq. (14) should be B(V)

We sincerely thank Reviewer 1 for the insightful comments and suggestions that have helped improve the clarity and quality of the final version. We have carefully considered all the remaining points raised and implemented the corresponding revisions in the manuscript.

Reviewer #2 (Remarks to the Author):

We thank the authors for their revisions and are satisfied with the responses provided. However, we hope the authors will carefully check the revised manuscript during proofing to ensure that all promised changes have been properly included.

In particular, the computational efficiency comparison (the N^2 scaling argument and hardware energy comparison) promised in the response cannot be found anywhere in the revised manuscript, and no GitHub repository URL is provided in either the manuscript or the response letter.
Reviewer #2 (Remarks on code availability):
No GitHub repository URL is provided.

We thank Reviewer 2 for the valuable comments, which helped improve the manuscript and bring it to its final form. We carefully proofread the manuscript and added a new subsection, “Computational Efficiency and Scaling,” to the Supplementary Material. We also included a link to the GitHub repository containing all the code required to reproduce the simulations and figures; the code is also available on OceanCode.

This paper presents an interesting result. Changing the current in adapting integrate-and-fire models from an adapting current (typically associated with an inward/inhibitory potassium current) to an outward (excitatory) current gives a model that can be used to implement working memory. The key observation is that changing the type of the current (by a simple sign change) means that the model can exhibit bistability: coexistence of a stable resting state and stable firing state. This allows the system to transition between resting and active states by short-term, sufficiently strong inputs, which the authors show can be used for working memory. The authors present three example models from the literature where this adjustment could be made and show a circuit implementation of one of them.

In general the organization and presentation of the paper is poor and doesn't seem of sufficient quality for Nature Communications at this stage. I have made specific notes below.

Section IIA presents a rather poorly justified back-of-the envelope calculation as to why models with the excitatory current have this bistability property. In particular, more justification and statement of underlying assumptions for eq.(10) are needed. (E.g., as shown later on the spiking solution, I_w periodically varies between two values, so what does I_w in eq. (10) represent? Minimum, maximum, mean?). Also how was the stability of the intersection points in Fig. 3 shown? The concluding paragraph (p. 10)

> "From this construction we conclude that the existence of a stable self-consistent self-excitation is a property of any neuron model where the activation function is convex or where the membrane current shows a saturation effect with the frequency, i.e., $I_w(f)$ is sub-linear."

is a vast overstatement.

Section IIB presents an analysis of one of the example models, which shows in a more methodical way why the bistabiity occurs, although some of the steps and methodology are not clear. In a sense this section makes the previous one redundant. All that is needed is to show bistability in the model and this can be done via phase plane and bifurcation analysis. A clear presentation of how this occurs for the eQIF model in the main body of the text and diagrams such as Fig. 3 and Fig. 5 for the other two models in the appendix, could then be followed up with a discussion of the situation for a general model.

The ideas could be strengthened if some discussion of how large (with respect to I_{ext}) the region of bistability the model needs to be in order for working memory to be viable. In Fig. 6 the region seems quite large. If parameters are varied so that this is smaller is the working memory still robust?

Section IIC presents a network version of the model and corresponding mean-field model. The key point seems to be that the network inherits the properties of the single neuron, i.e., it can store a single memory. I don't see why this is interesting. If the network were able to store many memories that might be interesting. In fact, contrary to the statment on p. 14 the reason many people study mean-field models is not because they replicate the behaviour of the single neuron but to show how networks can exhibit new behaviour not possible in the single neuron model.

Section IID presents experiments of a circuit implementation of. This is very interesting, but they should have mentioned that hardware schematic is in Appendix C.

Other comments, typos and clarifications

- p.8 line 145 refs to left panel of Figs 1 and 2 but they have no left panel.
- p.6, line 130. I_{KCa} is not excitatory current. Since it is a potassium current it slows down or prevents spiking.
- p.10 How is red curve in Fig 3 computed? The frequency formula is exact for steady state firing frequency of QIF model, so I don't understand why you say the formula provides a "good fit" of the FI curve. Might be better stated the numerical method that is providing a good replication of the exact formula.
- p.11 It is not clear why the paper brings up the fact that the model changes from type I to type II depending on whether the applied current is increasing or decreasing. Is/how is this relevant?
- p.11 Guttman, Lewis & Rinzel (1980) J. Physiol. 305: 377-395 show a figure of an experiment on the squid giant axon that is very similar to Fig. 4(a). It is worth citing as an example of a neuron that exhibits bistability.
- p.13 What are the numerical packages/methods used to generate Figs. 5 and 6? I assume that the variability in the height of the APs in Fig. 6 is due to numerical method? How were the limit cycles in Fig. 6 generated?
- p.13 Fig. 5 would be improved by showing trajectories for two initial conditions illustrating the bistability.
- p.13 "Obtain persistence in large ..." persistence of what?
- p.14 Don't need intrinsically bursting neurons to get population bursts. Quite the opposite. The key point of several studies is that population bursting arises when intrinsically spiking neurons are coupled together, i.e., it is a network induced phenomenon.
- p.16 eq. (13) $g_{syn,i}$ should be g_{syn}
